# Global reduction of snow cover in ski areas under climate change

**DOI:** 10.1371/journal.pone.0299735

**Published:** 2024-03-13

**Authors:** Veronika Mitterwallner, Manuel Steinbauer, Gregor Mathes, Anna Walentowitz

**Affiliations:** 1 Sport Ecology, Bayreuth Center of Ecology and Environmental Research (BayCEER) & Bayreuth Center of Sport Science (BaySpo), University of Bayreuth, Bayreuth, Germany; 2 Paleontological Institute and Museum, University of Zurich, Zurich, Switzerland; 3 Department of Biogeography, Bayreuth Center of Ecology and Environmental Research (BayCEER), University of Bayreuth, Bayreuth, Germany; ICIMOD: International Centre for Integrated Mountain Development, NEPAL

## Abstract

Ongoing climate change substantially alters snowfall patterns with severe but diverging consequences for global ski areas. A global assessment as well as the investigation of potential implications for mountain ecosystems is currently lacking. We quantify future trends in natural snow cover days under different climate change scenarios until 2100 in seven major global skiing regions and discuss implications for mountainous biodiversity by analysing how natural snow cover days relate to regional human population density. Within all major skiing regions, snow cover days are projected to decrease substantially under every assessed climate change scenario. Thirteen percent of all current ski areas are projected to completely lose natural annual snow cover and one fifth will experience a reduction of more than 50% by 2071–2100 relative to historic baselines. Future skiable areas will concentrate in less populated areas, towards continental regions and inner parts of the mountain ranges. As skiable areas will be located at greater distances to highly populated areas in the future, we expect an expansion of infrastructure and increasing intervening actions (i.e., artificial snowmaking, slope grooming) to prolong snow duration. Our results are concerning for both the recreational and economic value of skiing as well as for mountain biodiversity since vulnerable high-altitude species might be threatened by space reductions with ski area expansion.

## Introduction

Climate change is negatively affecting snow reliability in ski areas all over the world. Concern about how skiable the future will be is rising amongst professional and amateur skiers and the skiing industry. The negative consequences of climate change in terms of declining snow depth and reduced snow duration are reported for many regions worldwide [1–3]. These findings are socio-economically and ecologically concerning. Skiing and its touristic value are of great importance for local economies [4]. In addition, biodiversity in mountainous areas is already heavily affected by global warming [5] and is likely to receive additional pressure by expanding ski areas and a concentration of skiers towards higher elevations. As area is compacting towards higher elevations, conflicts between skiing and alpine biodiversity are likely to increase with the reduction of skiable areas towards higher elevations. Despite this ubiquitous potential for space conflicts between nature conservation and downhill skiing, global assessments of snow cover conditions in ski areas under climate change is lacking.

The economic recreational value of ski areas has raised interest in future projections of snow cover and snow duration in times of climate change. Regional studies assessing future snow reliability project decreasing snow cover days (scd), reduced ski season length and overall reduced snow reliability particularly at low elevations [1, 6–10]. In the European Alps, a ‘Christmas-Easter shift’ will be likely as optimal ski days are predicted to shift towards the end of the ski season [11]. Generally, research investigating the effects of climate change on snow reliability is biased towards Europe, North America and Australia and is targeting mostly small-scale assessments. This reflects the economic importance of skiing, with the European Alps having the largest global ski market, followed by North America [12]. Technical improvements and management strategies, such as artificial snowmaking, are widely used to counteract snow scarcity and to ensure economic success of ski areas, which is marked by the 100-day-rule [13]. However, artificial snowmaking will presumably not be a sufficient compensation measure under a severe climate change scenario [8]. Other adaptation strategies like slope contouring, landscaping, the protection of glaciers on existing skiable terrain as well as the development of new ski areas at, for instance, north facing and higher elevated slopes [14] are in addition commonly considered as threats to nature and the unique mountainous biodiversity [15]. Associated risks are likely to increase with changing snow availability due to climate change.

Besides the importance of high-elevation mountainous systems for the skiing sector, they also harbour a unique biodiversity, including numerous endemic and vulnerable species [16]. These systems are threatened by both climate change [5, 17] and by changes in tourism [18] as a response to climate change. Spatial conflicts between potential areas suitable for skiing and biodiversity conservation, especially of high-elevation species, can intensify with climate change. An uplifting of both, suitable habitats for species, as well as areas with sufficient snow availability for ski areas, is expected under ongoing climate change [16]. The spatial conflict will be exacerbated by the decline in available area towards higher elevated mountain areas. This effect potentially includes tendencies of ski areas expanding into less populated and yet naturally protected or less disturbed mountain areas at high elevations. Such a spatial focus of ski areas towards remote systems would most likely result in the expansion of infrastructure and thus habitat degradation as well as the loss of mountainous flora and fauna. Negative effects of ski areas on nature are widely investigated and range from changes of soil properties due to grooming and snowing activities to habitat fragmentation [19–22].

Despite the global potential for an intensification of spatial conflicts between downhill skiing and nature conservation with global warming, studies targeting the assessment of the distribution of future ski areas mostly focus on regional scales and some major mountain areas have not yet been considered. Such assessments have so far been limited by the lack of global-scale and fine-resolution projections of snow cover days (scd) as well as openly available geo-information on ski areas. Since data availability is rapidly growing, this study is the first to combine open-source information on global geo-locations of ski areas with openly available global snow cover day projections. Our approach enables assessing the global patterns of altering ski area distributions by identifying future trends (2011–2100) in natural snow cover days for ski areas worldwide. We focus on seven major mountainous skiing regions, namely, European Alps, Andes, Appalachian Mountains, Australian Alps, Japanese Alps, Southern Alps, and Rocky Mountains under the emissions scenarios SSP1-2.6, SSP3-7.0 and SSP5-8.5. Resulting range contractions and the change in annual snow cover days of ski areas will be assessed. We hypothesize that (i) annual snow cover days in ski areas will decrease globally and under all SSP scenarios and that (ii) optimal climate for ski areas will be located in less populated areas in the future. On this basis, we will discuss consequences for alpine biodiversity and arising conflicts between ski areas and nature protection.

## Methods

### Data acquisition

#### Snow cover days

Global raster data on natural annual snow cover days (scd) at a resolution of 30 arc sec for a historic baseline (1950–2010), the present (2011–2040), immediate future (2041–2070) and near future (2071–2100) based on a ISIMIP3b model were selected from the CHELSA (Climatologies at high resolution for earth’s land surface areas) database [23, 24]. The projections are based on GFDL’s Earth System Model Version 4 (GFDL-ESM4, ISIMIP3b, www.isimip.org) for SSP1-2.6 (low), SSP3-7.0 (high) and SSP5-8.5 (very high) emissions scenarios. GFDL-ESM4 is a chemistry-carbon-climate Earth system model focusing on Earth system interactions with a doubled horizontal resolution [25]. The projected snow cover days on a high resolution are based on a temperature model using statistical downscaling and a precipitation model which consists of various orographic predictors [24].

#### Global ski areas

We retrieved information on the global distribution of ski areas from OpenStreetMap via the overpass API (overpass-turbo.eu) using the tag search term “piste:type = downhill” (downloaded 30.06.2020). The tagged ski areas are the result of openly available collaboratively generated citizen science data. Data quality was ensured by combining information on the occurrence of ski areas from OpenStreetMap with global projections of annual scd from 2005 to 2018. For 2005 to 2013, raster files on annual scd were downloaded from the CHELSA Database [26] and annual scd from 2014 to 2018 were calculated using Saga GIS Version 2.3.2 (snow cover tool) using monthly temperature and precipitation data at a resolution of 30 arc sec [26]. All indicated ski areas were categorized according to the number of annual scd from 2005 to 2018 (from 0 = no snow days to 365 = snow all year long). Those falling into pixels of zero scd were manually checked and subsequently eliminated from the dataset in cases where they were incorrectly tagged as ski area or turned out to be indoor skiing centres and dry or artificial ski slopes. The occurrence of ski areas was translated into a global presence-absence raster on the resolution of the annual scd data. From this global distribution of ski areas, we extracted seven major mountain regions, namely the Andes, Appalachian Mountains, Australian Alps, European Alps, Japanese Alps, Rocky Mountains, and the Southern Alps (New Zealand). The European Alps account for 69% of all pixels with documented downhill skiing, followed by the Rocky Mountains that sum up to 15%. Japanese Alps and the Appalachian Mountains sum up to 7% each, the Andes and Australia to 1% each, and Southern Alps accounts for 0.5% of the data.

#### Population density

To assess how global ski areas are currently distributed relative to population density, data on global population density from 2020 was retrieved at a resolution of 30 arc sec from the Socioeconomic Data and Applications Centre (SEDAC) as part of NASA’s Earth Observing System Data and Information System (EOSDIS) as a geo-referenced raster file [27].

### Analysis

For every pixel for which ski areas have been recorded within the seven mountain regions, we extracted the number of annual natural snow cover days for the periods 1981–2010 (historical), 2011–2040 (present), 2041–2070 (immediate future) and 2071–2100 (near future) given a very high (SSP5-8.5), high (SSP3-7.0) and low (SSP1-2.6) emissions scenario. A nonparametric Kruskal-Wallis test and a Pairwise Wilcoxon test with Bonferroni correction was implemented to detect differences in annual scd between periods [28] and the results were visualized using underlying data distributions. The percent change of future annual scd relative to a historic baseline of annual scd was depicted via change maps (% change in annual scd) within all seven mountain regions. To assess the potential concentration of future ski areas towards less populated areas in high altitudes, we plotted annual scd for all ski areas combined and separated into regions with low (<25% percentile), medium (>25% and <75% percentile) and high (>75% percentile) population density. Percentiles were based on single regions. We calculated the percent change in annual scd for future periods relative to historic numbers and differentiated between low, medium, and high population density in the ski areas to evaluate the concentration effect of future reliable ski areas into less populated areas.

Analysis and figures were implemented for all emissions scenarios (SSP1-2.6, SSP3.-7.0, SSP5.-8.5), since there is contrasting evidence which scenario best tracks current emissions trajectories [29, 30]. While we compare the three scenarios in our results, we focus on outlining the results based on the high emissions scenario (SSP3-7.0) as warming is more pronounced in terrestrial systems compared to the Earth’s average, and mountain systems are particularly sensitive [31]. Detailed results on the low and very high emissions scenarios are reported in the supplementary information (S1–S5 Tables and S1–S5 Figs). All analysis and visualisation were conducted via R version 4.0.5 (R Core Team 2021) using the package raster [32] and elevatr [33].

## Results

**Fig 1:**
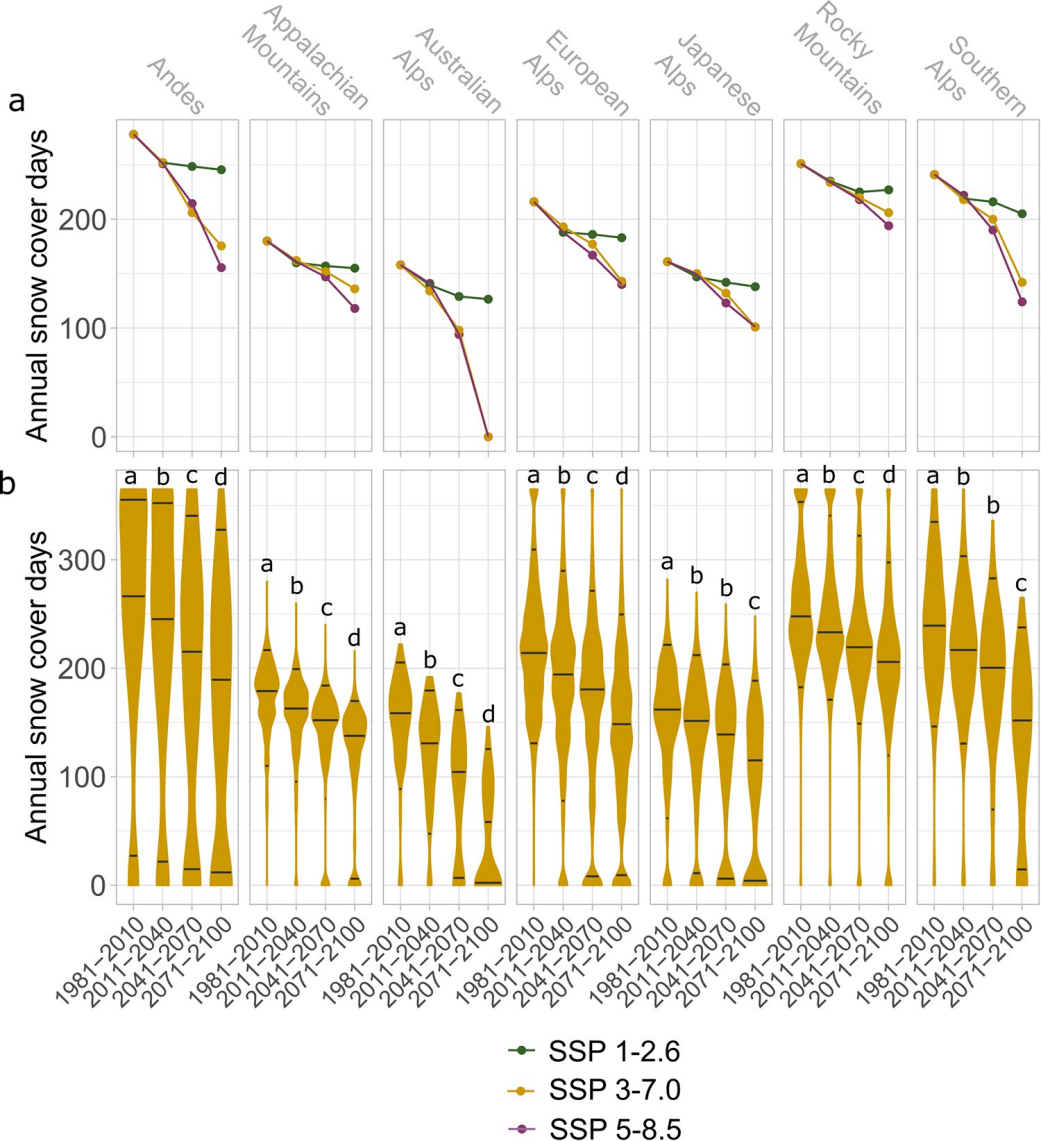
Annual snow cover days between historical times (1981–2011) and the end of the century (2071–2100) in current ski areas of the seven major mountain regions worldwide as a) trends for low (SSP1-2.6, green), high (SSP3-7.0, yellow) and very high (SSP5-8.5, violet) emissions scenarios and as b) violin plots under a high emissions scenario. The median and the 5%;95% confidence intervals are indicated by black lines and the width and length of the violins show the total number of ski areas. Letters above the violin plots indicate significant differences between groups (Kruskal-Wallis test, Pairwise Wilcoxon test with Bonferroni correction, e.g. a differs significantly from b, c, and d).

Annual snow cover days in the seven major mountain areas with downhill skiing will significantly decrease worldwide with climate change under a very high, high, and low emissions scenario (Fig 1, S1 and S2 Figs). For all currently recorded ski areas, annual scd will decrease substantially under a high emissions scenario (Fig 1; S1 Table). Mean annual scd will decline by 43% (Andes), 37% (Appalachian), 78% (Australian Alps), 42% (European Alps), 50% (Japanese Alps), 23% (Rocky Mountains) and 51% (Southern Alps) by 2071–2100 relative to historic times (S2 Table). On average, annual scd in many ski areas of the main global mountain regions will approach the threshold of 100 annual scd by the end of this century (Andes: 153 (-97.6) scd, European Alps: 137 (-80.3) scd, Southern Alps: 128 (-112.3) scd) and the Appalachian Mountains: 116 (-57.3). In the Japanese Alps snow cover duration is projected to cross this threshold with 86 (-64.4) annual scd by 2071–2100. With 81 (-69.2) annual scd by 2041–270 and 38 (-111.5) by 2071–2100, the average annual scd in the Australian Alps will reach this threshold sooner (S1 and S3 Tables). Despite similar losses in scd (-56.4), the Rocky Mountains will remain relatively snow reliable with on average 202 annual scd by the end of this century.

Annual scd in global ski areas will similarly decrease under a low and substantially decrease under a very high emissions scenario by the end of this century (S4 and S5 Tables, S1 and S2 Figs). In the Andes and the Southern Alps, however, annual scd will decrease more slowly in a low emissions scenario. In addition, in a low emissions scenario, no regions will on average fall below 100 annual scd, whereas the Australian Alps, the Appalachian Mountains and the Japanese Alps will fall beneath this threshold under a very high emissions scenario (S4 and S5 Tables). In general, all projections show an overall reduction of ski areas and a substantial increase in present ski areas with zero annual scd for future periods.

**Fig 2:**
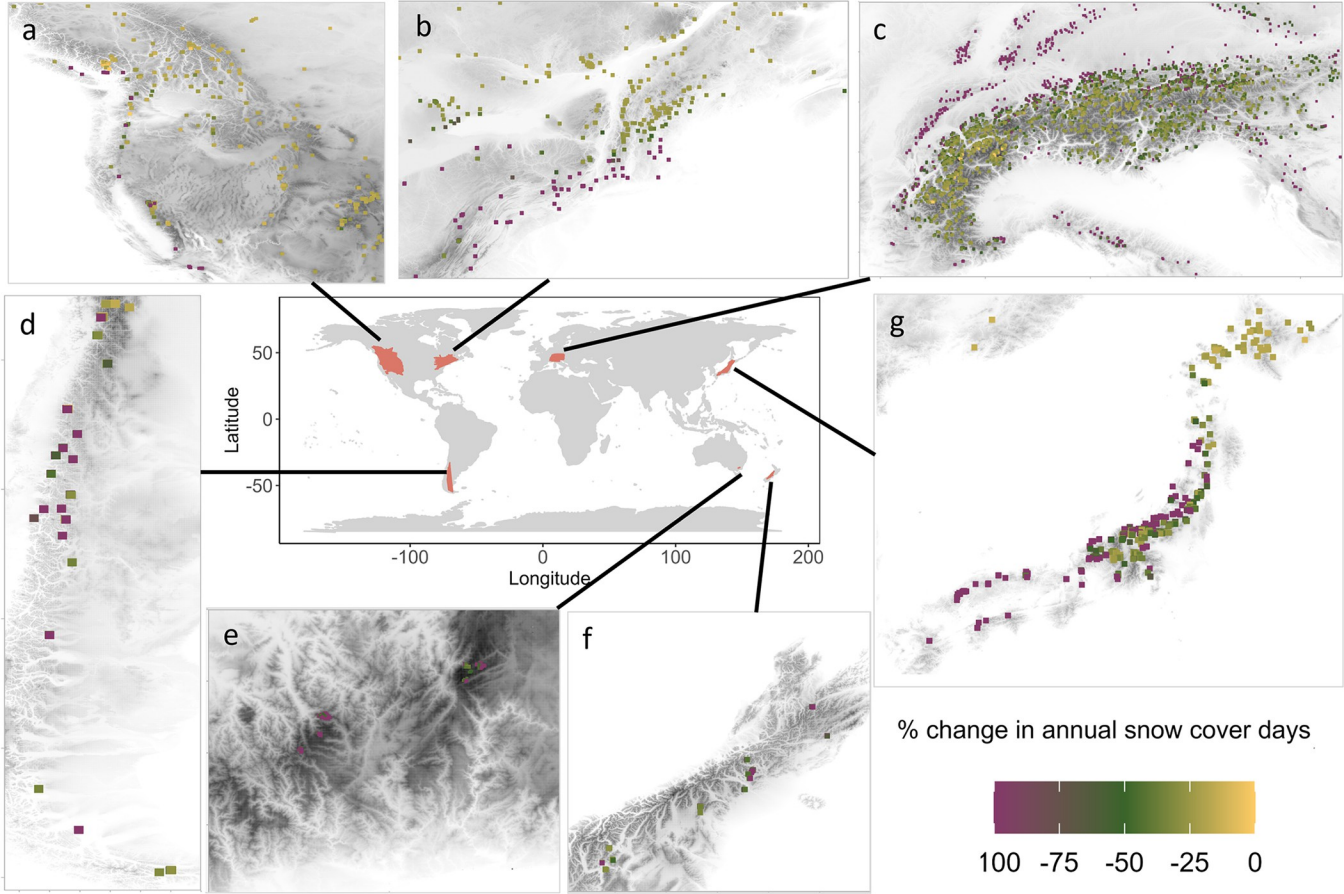
Major global mountainous regions (a) Rocky Mountains b) Appalachian Mountains c) European Alps d) Andes e) Australian Alps f) Southern Alps g) Japanese Alps) with ski areas and an underlying elevation gradient (grey, details in S10–S16 Figs). Colouring indicates the percent change in annual snow cover days in each ski area in the future 2071–2100 compared to historical (1981–2010) snow cover days under a high (SSP3-7.0) emissions scenario.

Globally, 13% of current ski areas will experience a 100% decrease in annual snow cover days by 2071–2100 and 20% will decrease between 50 and 100% (Fig 2). Under a high emissions scenario, ski areas in the Southern Hemisphere (Andes, Australian Alps, Southern Alps) as well as the Japanese Alps and the Appalachians will be most severely affected by climate change. Eighteen percent of the Andean ski areas will decrease by 100% and another 31% by 50% or more. Similar reductions will be reached in the Appalachian Mountains and the Japanese Alps, where 14% and 17% of the ski areas will decrease completely and around one fourth will decline by more than 50%. Worse reductions in annual scd will occur in the Southern Alps with 30% and the Australian Alps with 78% of the ski areas declining in annual scd by more than 50%. Generally, ski areas with secure future snow conditions (0 to -50% change in scd) in the European Alps, Japanese Alps and Southern Alps will considerably concentrate towards central and higher elevated parts of mountain ranges (Fig 2). Annual snow cover days of ski areas in the southern and coastal mountain areas of North America will be more affected by climate change and decrease by 50% to 100%. More continental and northern ski areas in the Rocky Mountains will have a less pronounced loss in annual scd (< 50%) (Fig 2). Under a very high emissions scenario these patterns are more pronounced (S3–S9 Figs).

**Fig 3:**
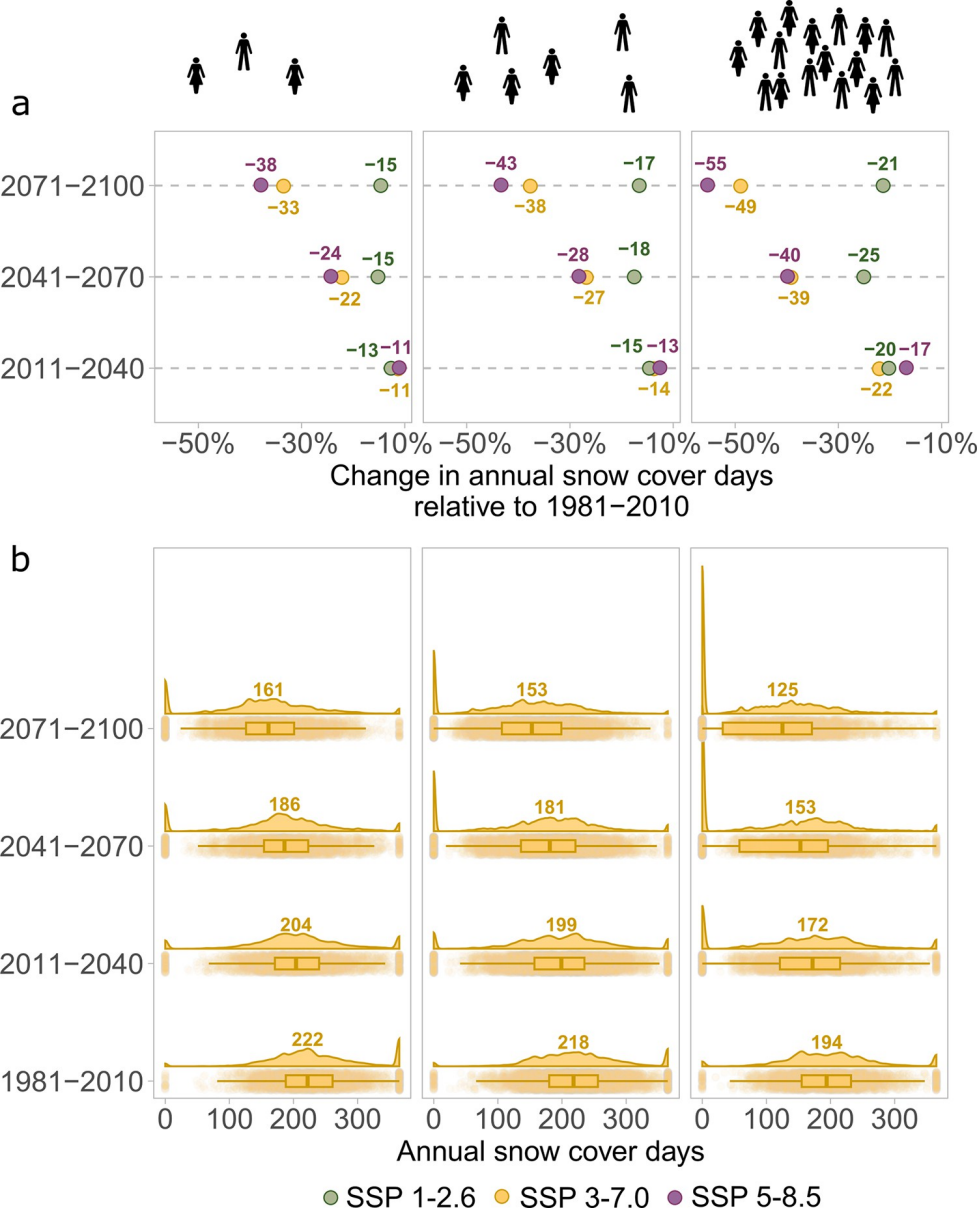
a) Percent change in annual snow cover days in future periods relative to 1981–2100 for all ski areas combined and separated into low (<25% percentile, left), medium (>25% and <75% percentile, middle) and high (>75% percentile, right) population density and for low (SSP1-2.6, green), high (SSP3-7.0, yellow) and very high (SSP5-8.5, violet) emissions scenarios. b) Historical, current, and future annual snow cover days for all ski areas, separated into areas with low, medium, and high population density for high (SSP3-7.0, yellow) emissions scenarios.

With ongoing climate change, annual scd of global ski areas in less populated areas will decrease less pronounced compared to medium and highly populated areas under a high (Fig 3), very high (S17 Fig) and low emissions scenario (S18 Fig). Ski areas in highly populated areas will be most affected by decreasing annual scd and are projected to decline by 55%/49% (very high/high emissions scenario) from on average 194 scd in 1981–2010 to 112/125 (very high/high emissions scenario) scd in the future. By 2041–2070, annual scd in highly populated areas will decline by 40%/39% to 142/153 annual scd on average. Decrease in annual scd will be less pronounced in intermediately populated areas with a decline of 43%/38% (218 to 146/153 mean annual scd) and poorly populated areas with a reduction of 38%/33% (222 to 156/161 mean annual scd) annual scd in the future (Fig 3 and S18 Fig). Overall, year-round snow cover in present ski areas will nearly disappear worldwide, whereas the number of ski areas with zero snow cover days will increase substantially. Most considerably, this will happen in highly populated areas (Fig 3B and S17, S18 Figs).

## Discussion

Within this century, ongoing climate change will globally lead to a substantial reduction in annual snow cover days in current ski areas under all emissions scenarios. Ski areas located in highly populated areas are predicted to be more affected by global warming and hence, ecological conflicts will become more likely as skiable areas will concentrate towards remote mountainous areas in the future.

This negative trend in global scd within ski areas is in line with regional studies [1, 6–10]. In addition, similar negative effects on global snow cover duration were observed between 2000 and 2018 based on remote sensing MODIS products, with decreases of up to 43 days in 78% of the global mountain areas [34]. Ongoing warming causes a shift from solid to liquid precipitation [35, 36] and precipitation will additionally decline under future climate conditions in many mountain regions such as the European Alps [37]. Hence, climate change affects snow depth and duration negatively [38]. Consequently, a substantial decrease in snow cover duration is projected by the end of this century, for instance, in Europe [7, 39]. Likewise, our results show that this substantial reduction in annual scd is not restricted to regions but can be detected globally. However, regarding the long-term effect of climate change on snow reliability in ski areas, high altitude ski areas of the European Alps and the Rocky Mountains are projected to be on average less affected than all other mountain skiing regions targeted in this study. These results are supported by existing regional literature, although projections vary within regions in dependence of local climatic conditions and elevation [1, 3, 7, 10, 40–42].

Since this study focuses on natural snow cover, the projections are only partly applicable for ski areas implementing technical snow management such as grooming, slope preparation or snowmaking [43]. Nevertheless, technical snowmaking in ski areas will have to be dramatically increased to compensate natural snow scarcity and to ensure economic profitability. The lack of snow is already problematic in many regions, with technical snowmaking increasing in importance for decades [14]. Predicted increases in the crucial amount of additional snowmaking under future conditions varies depending on regional conditions and elevation [40, 42]. However, this measure is limited by the availability of fresh-water resources and by ambient temperature and costly due to high energy consumption [44, 45]. Particularly low altitude ski areas are more affected by climate change [6, 46] and artificial snow production will not compensate for the missing snow due to high economic costs, high resource demands and a lack of cold temperatures in the future [6, 40, 41, 47]. In addition, technical snow production can also have considerable ecological implications, like nutrient increase [48], change of soil properties [49] or impacts on plant phenology [50].

We project a considerable increase in ski areas completely lacking snow in the future. Thirteen percent of all current ski areas will have a 100% decrease in annual snow cover days and one fifth will experience a reduction of 50% to 100% scd in 2071–2100 relative to a historic baseline under a high emissions scenario. In accordance, 80% snow reduction is projected globally under SSP5-8.5, although reductions are smaller in higher altitudes [51]. Moreover, we illustrate in this study that outward parts of mountainous areas and coastal ski areas are most affected by global warming. Hence, in the future, present ski areas will concentrate towards continental regions and central parts of the mountain ranges in higher elevations. The highest climate sensitivity was found for midlatitudinal coastal mountains in western North America with maritime climates, whereas areas with continental interior climates were less sensitive to climate change in regard of snow cover reliability [52]. In the Northeast of the US, viable ski areas are projected to contract northwards [53], which is supported by our results. Accordingly, in the European Alps, a substantial reduction of snow water equivalent [9] is resulting in an upward shift of snow reliability [37]. Furthermore, the probability of optimal ski days is higher in the inner Alps and higher regions in the immediate future [11]. Besides the number of optimal ski days, the 100-day-rule [13] is used as an indicator for snow reliability and hence, the economic profitability of ski resorts. Our results indicate that a substantial proportion of present ski areas will globally fall below this threshold (Fig 2B). Another important skiing market, which was recently growing tremendously, is China. This country has not been included in this study as data on Chinese ski areas is lacking in OpenStreetMap. However, a considerable shortening of the ski season length in most low altitude ski areas is similarly projected for China and the 100-day-rule will only be reached by 65% of Chinese ski resorts by the mid-century [40].

In this study, we used annual natural scd to identify global patterns of snow reliability in ski areas. Hence, while enabling the comparison of scd in mountain regions between future time periods and historic values, this study is limited in showing differences in spatial or temporal dynamics on a finer scale. Ski areas are characterized by extreme climate conditions [54], which is why other approaches are more useful when evaluating snow cover area more locally [55–57]. In addition, we showed the results for three different global emissions pathways, while focusing on a high emissions scenario. Although there is contrasting evidence in the literature on which emissions pathways tracks current trajectories best [29–31], the very high emissions scenario (SSP5-8.5) is unlikely to be met. However, since mountain systems are particularly sensitive to climate warming, projecting the results for the high and very high emissions scenario is likely to be more relevant compared to other systems.

Our results indicate space contractions in present ski areas. Overall, less ski areas will remain snow reliable under future climate conditions and hence, skiable areas will be concentrated to spatially restricted higher elevations. Additionally, we show that areas in highly populated areas are most affected by climate change. Thus, future ski areas will concentrate towards less populated areas, although it is important to mention that this analysis relies on a constant population density, which is highly unlikely in the future. In fact, population density is projected to increase for mountain areas under SSP3 [58]. Such an increase in population density could imply more pressure on alpine ecosystems in the long-term, in addition to the threat of ski area expansions in high-altitude ecosystems. This trend to expand towards higher and more remote areas is not yet quantified, but well demonstrated in our results. Consequently, an increase in transportation efforts is likely as people who were formerly skiing in near and low altitude areas will have to travel towards the inner parts of the mountain ranges and towards higher altitudes. The combination of a concentration of ski areas towards higher altitudes and the resulting expansion of infrastructure to enable access to skiable areas is additionally of concern for mountain biodiversity. High altitudes are known to be a biodiversity hotspot and serve as refuge for vulnerable species, which might be directly affected by the expanse of ski areas and the increase in ski tourists. Alpine plants are known to only slowly recover from disturbances [20]. Moreover, indirect effects on alpine species are likely. For instance, ski areas are associated with an anticipation of plant phenology [50], potentially inducing a mismatch between plant blooming and herbivore activity [59]. Biodiversity conservation should be aware of the potential trajectories, not only of climate warming per se, but also of the implications of climate change for skiing. We expect conflicts between nature and skiers to increase with ongoing climate change.

We project substantially reduced snow reliability in current ski areas on a global scale and an overall reduced number of skiable areas worldwide. Additionally, we show that coastal, low-latitudinal regions and highly populated areas, as well as outer and lower parts of the mountain ranges, are more affected by climate change. Therefore, we conclude that whilst the ski market is not projected to decrease in the near future, higher elevated and less populated ski areas will be expanding in size and infrastructure. These developments are alerting for the nature in mountain areas, as spatial compromises will most likely have to be made between future skiable areas and space for conservation of alpine biodiversity.

## Supporting information

S1 Table Annual snow cover days (mean, median, 5% and 95% confidence interval) per time period and in seven major mountain skiing areas under a high (SSP3-7.0) emissions scenario. (PDF)

S2 Table Annual snow cover days (mean, median, 5% and 95% confidence interval) per time period and in seven major mountain skiing areas under a very high (SSP5-8.5) emissions scenario. (PDF)

S3 Table Annual snow cover days (mean, median, 5% and 95% confidence interval) per time period and in seven major mountain skiing areas under a low (SSP1-2.6) emissions scenario. (PDF)

S4 Table Mean and statistic differences between historical (1981–2011), present (2011–2040) and future (I: 2041–2070, II: 2071–2100) annual snow cover days in seven major skiing regions worldwide based on a Kruskal-Wallis-Test and a Pairwise Wilcoxon test with Bonferroni correction (alpha = 0.05) for very high, high and low emissions scenarios. Significant relationships (α = 0.05) are indicated in bold letters.
(PDF)

S5 Table Percent change in annual snow cover days between a historic base line (1981–2011), the present (2011–2040) and the future (I: 2041–2070, II: 2071–2100) in seven major skiing regions worldwide. Confidence intervals are given (CI 5;95). Data for very high, high and low emissions scenarios is given.
(PDF)

S1 Fig Violin plots of historical (1981–2011), present (2011–2040) and future (2041–2070; 2071–2100) annual snow cover days in seven major skiing regions worldwide under a very high emissions scenario (SSP5-8.5 scenario). The median and the 5%;95% confidence intervals are indicated by black lines. Letters above the violin plots indicate significant differences between groups (Kruskal-Wallis test, Pairwise Wilcoxon test with Bonferroni correction, e.g. a differs significantly from b, c, and d*)*.
(TIF)

S2 Fig Violin plots of historical (1981–2011), present (2011–2040) and future (2041–2070; 2071–2100) annual snow cover days in seven major skiing regions worldwide under a low emissions scenario (SSP1-2.6). The median and the 5%;95% confidence intervals are indicated by black lines. Letters above the violin plots indicate significant differences between groups (Kruskal-Wallis test, Pairwise Wilcoxon test with Bonferroni correction, e.g. a differs significantly from b, c, and d*)*.
(TIF)

S3 Fig European Alps with ski areas and an underlying elevation layer (grey). Colouring indicates the percent change in annual snow cover days in each ski area in the future 2071–2100 compared to historical (1981–2010) snow cover days under very high emissions (SSP5-8.5) scenario.
(TIF)

S4 Fig Andes with ski areas and an underlying elevation layer (grey). Colouring indicates the percent change in annual snow cover days in each ski area in the future 2071–2100 compared to historical (1981–2010) snow cover days under very high emissions (SSP5-8.5) scenario.
(TIF)

S5 Fig Appalachian Mountains with ski areas and an underlying elevation layer (grey). Colouring indicates the percent change in annual snow cover days in each ski area in the future 2071–2100 compared to historical (1981–2010) snow cover days under very high emissions (SSP5-8.5) scenario.
(TIF)

S6 Fig Australian Alps with ski areas and an underlying elevation layer (grey). Colouring indicates the percent change in annual snow cover days in each ski area in the future 2071–2100 compared to historical (1981–2010) snow cover days under very high emissions (SSP5-8.5) scenario.
(TIF)

S7 Fig Japanese Alps with ski areas and an underlying elevation layer (grey). Colouring indicates the percent change in annual snow cover days in each ski area in the future 2071–2100 compared to historical (1981–2010) snow cover days under very high emissions (SSP5-8.5) scenario.
(TIF)

S8 Fig Southern Alps with ski areas and an underlying elevation layer (grey). Colouring indicates the percent change in annual snow cover days in each ski area in the future 2071–2100 compared to historical (1981–2010) snow cover days under very high emissions (SSP5-8.5) scenario.
(TIF)

S9 Fig Rocky Mountains with ski areas and an underlying elevation layer (grey). Colouring indicates the percent change in annual snow cover days in each ski area in the future 2071–2100 compared to historical (1981–2010) snow cover days under very high emissions (SSP5-8.5) scenario.
(TIF)

S10 Fig European Alps with ski areas and an underlying elevation layer (grey). Colouring indicates the percent change in annual snow cover days in each ski area in the future 2071–2100 compared to historical (1981–2010) snow cover days under high emissions (SSP3-7.0) scenario.
(TIF)

S11 Fig Andes with ski areas and an underlying elevation layer (grey). Colouring indicates the percent change in annual snow cover days in each ski area in the future 2071–2100 compared to historical (1981–2010) snow cover days under high emissions (SSP3-7.0) scenario.
(TIF)

S12 Fig Appalachian Mountains with ski areas and an underlying elevation layer (grey). Colouring indicates the percent change in annual snow cover days in each ski area in the future 2071–2100 compared to historical (1981–2010) snow cover days under high emissions (SSP3-7.0) scenario.
(TIF)

S13 Fig Australian Alps with ski areas and an underlying elevation layer (grey). Colouring indicates the percent change in annual snow cover days in each ski area in the future 2071–2100 compared to historical (1981–2010) snow cover days under high emissions (SSP3-7.0) scenario.
(TIF)

S14 Fig Japanese Alps with ski areas and an underlying elevation layer (grey). Colouring indicates the percent change in annual snow cover days in each ski area in the future 2071–2100 compared to historical (1981–2010) snow cover days under high emissions (SSP3-7.0) scenario.
(TIF)

S15 Fig Southern Alps with ski areas and an underlying elevation layer (grey). Colouring indicates the percent change in annual snow cover days in each ski area in the future 2071–2100 compared to historical (1981–2010) snow cover days under high emissions (SSP3-7.0) scenario.
(TIF)

S16 Fig Rocky Mountains with ski areas and an underlying elevation layer (grey). Colouring indicates the percent change in annual snow cover days in each ski area in the future 2071–2100 compared to historical (1981–2010) snow cover days under high emissions (SSP3-7.0) scenario.
(TIF)

S17 Fig Historical and future annual snow cover days for all skiing areas combined and separated into low, medium and high population density. The figure is based on a very high (SSP5-8.5) emissions scenario.
(TIF)

S18 Fig Historical and future annual snow cover days for all skiing areas combined and separated into low, medium and high population density. The figure is based on a low (SSP1-2.6) emissions scenario.
(TIF)
