# Supplementary material for: Global reduction of snow cover in ski areas under climate change
Source: PLoS One. 2024 Mar 13;19(3):e0299735. doi: 10.1371/journal.pone.0299735 (PMC10936838; doi:10.1371/journal.pone.0299735)
Supplement: S1 Table — (PDF) [file pone.0299735.s001.pdf]

Region	Period	mean	median	CI (5%;95%)
**All regions**	Historical	216	214	124;344
	Present	189	193	28;305
	Future I	164	177	0;281
	Future II	141	149	0;258
**Andes**	Historical	251	278	0;365
	Present	226	252	0;365
	Future I	181	206	0;365
	Future II	153	176	0;322
**Appalachian**	Historical	174	180	110;216
	Present I	156	162	90;198
	Future I	139	152	0;182
	Future II	116	136	0;167
**Australian Alps**	Historical	150	158	60;201
	Present	121	134	0;179
	Future I	81	98	0;153
	Future II	38	0	0;117
**European Alps**	Historical	218	216	132;328
	Present	187	193	0;295
	Future I	160	177	0;270
	Future II	137	143	0;248
**Japanese Alps**	Historical	151	161	0;219
	Present	134	150	0;209
	Future I	108	132	0;196
	Future II	86	101	0;180
**Rocky Mountains**	Historical	258	251	184;365
	Present	242	234	171;365
	Future I	223	220	143;336
	Future II	202	206	63;303
**Southern Alps**	Historical	240	241	153;348
	Present	213	218	132;300
	Future I	189	200	26;276
	Future II	128	142	0;231
