# Supplementary material for: Global reduction of snow cover in ski areas under climate change
Source: PLoS One. 2024 Mar 13;19(3):e0299735. doi: 10.1371/journal.pone.0299735 (PMC10936838; doi:10.1371/journal.pone.0299735)
Supplement: S2 Table — (PDF) [file pone.0299735.s002.pdf]

| Region | Period | mean | median | CI (5%;95%) |
|---|---|---|---|---|
| **All regions** | Historical | 216 | 214 | 124;344 |
| | Present | 192 | 189 | 87;309 |
| | Future I | 161 | 169 | 0;279 |
| | Future II | 131 | 142 | 0;253 |
| **Andes** | Historical | 251 | 278 | 0;365 |
| | Present | 224 | 251 | 0;365 |
| | Future I | 191 | 215 | 0;365 |
| | Future II | 138 | 156 | 0;309 |
| **Appalachian** | Historical | 174 | 180 | 110;216 |
| | Present I | 153 | 161 | 80;196 |
| | Future I | 128 | 147 | 0;178 |
| | Future II | 94 | 118 | 0;156 |
| **Australian Alps** | Historical | 150 | 158 | 60;201 |
| | Present | 128 | 141 | 0;180 |
| | Future I | 76 | 94 | 0;152 |
| | Future II | 20 | 0 | 0;105 |
| **European Alps** | Historical | 218 | 216 | 132;328 |
| | Present | 190 | 188 | 92;297 |
| | Future I | 158 | 167 | 0;272 |
| | Future II | 129 | 140 | 0;247 |
| **Japanese Alps** | Historical | 151 | 161 | 0;219 |
| | Present | 136 | 150 | 0;206 |
| | Future I | 104 | 123 | 0;191 |
| | Future II | 83 | 101 | 0;175 |
| **Rocky Mountains** | Historical | 258 | 251 | 184;365 |
| | Present | 242 | 234 | 165;365 |
| | Future I | 219 | 218 | 133;329 |
| | Future II | 182 | 194 | 0;296 |
| **Southern Alps** | Historical | 240 | 241 | 153;348 |
| | Present | 216 | 222 | 138;301 |
| | Future I | 181 | 190 | 0;273 |
| | Future II | 110 | 124 | 0;233 |
