# Supplementary material for: Global reduction of snow cover in ski areas under climate change
Source: PLoS One. 2024 Mar 13;19(3):e0299735. doi: 10.1371/journal.pone.0299735 (PMC10936838; doi:10.1371/journal.pone.0299735)
Supplement: S3 Table — (PDF) [file pone.0299735.s003.pdf]

Region	Period	mean	median	CI (5%;95%)
**All regions**	Historical	216	214	124;344
	Present	188	188	68;301
	Future I	182	185	54;293
	Future II	184	183	74;295
**Andes**	Historical	251	278	0;365
	Present	227	252	0;365
	Future I	226	249	0;365
	Future II	217	246	0;365
**Appalachian**	Historical	174	180	110;216
	Present I	151	160	77;193
	Future I	145	157	54;189
	Future II	142	155	26;188
**Australian Alps**	Historical	150	158	60;201
	Present	125	140	0;184
	Future I	111	129	0;172
	Future II	108	127	0;173
**European Alps**	Historical	218	216	132;328
	Present	185	188	72;287
	Future I	181	186	62;284
	Future II	184	183	87;285
**Japanese Alps**	Historical	151	161	0;219
	Present	131	147	0;206
	Future I	122	142	0;198
	Future II	122	138	0;198
**Rocky Mountains**	Historical	258	251	184;365
	Present	242	235	166;365
	Future I	229	225	149;351
	Future II	231	227	152;346
**Southern Alps**	Historical	240	241	153;348
	Present	214	219	137;293
	Future I	211	216	128;299
	Future II	201	205	112;284
