# Supplementary material for: Global reduction of snow cover in ski areas under climate change
Source: PLoS One. 2024 Mar 13;19(3):e0299735. doi: 10.1371/journal.pone.0299735 (PMC10936838; doi:10.1371/journal.pone.0299735)
Supplement: S4 Table — Significant relationships (α = 0.05) are indicated in bold letters. (PDF) [file pone.0299735.s004.pdf]

Region	Timely comparison	SSP5-8.5		SSP3-7.0		SSP1-2.6	
		mean	p-value	mean	p-value	mean	p-value
**All regions**	Historical – present	**-24.5**	**<0.001**	**-26.6**	**<0.001**	**-28.3**	**<0.001**
	Historical –future I	**-55.0**	**<0.001**	**-51.6**	**<0.001**	**-34.3**	**<0.001**
	Historical – future II	**-85.0**	**<0.001**	**-74.6**	**<0.001**	**-32.5**	**<0.001**
	Present – future I	**-30.5**	**<0.001**	**-25.0**	**<0.001**	**-6.0**	**<0.001**
	Present – future II	**-60.5**	**<0.001**	**-47.9**	**<0.001**	**-4.1**	**<0.001**
	Future I – future II	**-30.0**	**<0.001**	**-22.9**	**<0.001**	1.8	1.0
**Andes**	Historical – present	-26.2	0.0536	-24.2	0.0595	-23.3	0.106
	Historical – future I	**-59.6**	**<0.001**	**-69.9**	**<0.001**	24.9	0.053
	Historical – future II	**-112.9**	**<0.001**	**-97.6**	**<0.001**	**-33.3**	**0.005**
	Present – future I	**-33.4**	**0.0023**	**-45.7**	**<0.001**	-1.6	1.0
	Present – future II	**-86.7**	**<0.001**	**-73.4**	**<0.001**	-10.0	1.0
	Future I – future II	**-53.3**	**<0.001**	**-27.8**	**0.0499**	-8.4	1.0
**Appalachian**	Historical – present	**-21.0**	**<0.001**	**-17.2**	**<0.001**	**-22.6**	**<0.001**
	Historical – future I	**-45.3**	**<0.001**	**-34.6**	**<0.001**	**-28.6**	**<0.001**
	Historical – future II	**-79.3**	**<0.001**	**-57.3**	**<0.001**	**-32.1**	**<0.001**
	Present – future I	**-24.3**	**<0.001**	**-17.4**	**<0.001**	**-6.0**	**<0.001**
	Present – future II	**-58.4**	**<0.001**	**-40.1**	**<0.001**	**-9.5**	**<0.001**
	Future I – future II	**-34.1**	**<0.001**	**-22.7**	**<0.001**	-3.5	0.07292
**Australian Alps**	Historical – present	**-21.8**	**<0.001**	**-28.6**	**<0.001**	**-24.5**	**<0.001**
	Historical – future I	**-73.7**	**<0.001**	**-69.2**	**<0.001**	**-39.3**	**<0.001**
	Historical – future II	**-129.9**	**<0.001**	**-111.5**	**<0.001**	**-42.2**	**<0.001**
	Present – future I	**-51.9**	**<0.001**	**-40.5**	**<0.001**	-14.8	0.27415
	Present – future II	**-108.1**	**<0.001**	**-82.9**	**<0.001**	**-17.7**	**0.04763**
	Future I – future II	**-56.2**	**<0.001**	**-42.3**	**<0.001**	-2.9	1.0
**European Alps**	Historical – present	**-27.7**	**<0.001**	**-30.8**	**<0.001**	**-32.4**	**<0.001**
	Historical – future I	**-60.1**	**<0.001**	**-57.3**	**<0.001**	**-36.5**	**<0.001**
	Historical – future II	**-88.2**	**<0.001**	**-80.3**	**<0.001**	**-33.8**	**<0.001**
	Present – future I	**-32.4**	**<0.001**	**-26.5**	**<0.001**	**-4.1**	**0.00264**
	Present – future II	**-60.6**	**<0.001**	**-49.5**	**<0.001**	**-1.4**	**<0.001**
	Future I – future II	**-28.1**	**<0.001**	**-22.9**	**<0.001**	2.7	1.0
**Japanese Alps**	Historical – present	**-14.8**	**<0.001**	**-17.2**	**<0.001**	**-20.1**	**<0.001**
	Historical – future I	**-46.6**	**<0.001**	**-42.6**	**<0.001**	**-29.0**	**<0.001**
	Historical – future II	**-67.7**	**<0.001**	**-64.4**	**<0.001**	**-29.2**	**<0.001**
	Present – future I	**-31.7**	**<0.001**	-25.4	**<0.001**	**-8.9**	**<0.001**
	Present – future II	**-52.8**	**<0.001**	**-47.2**	**<0.001**	**-9.1**	**<0.001**
	Future I – future II	**-21.1**	**<0.001**	**-21.8**	**<0.001**	0.2	1.0
**Rocky Mountains**	Historical – present	**-16.1**	**<0.001**	**-16.1**	**<0.001**	**-16.5**	**<0.001**
	Historical – future I	**-38.9**	**<0.001**	**-35.1**	**<0.001**	**-29.7**	**<0.001**
	Historical – future II	**-75.8**	**<0.001**	**-56.4**	**<0.001**	**-27.3**	**<0.001**
	Present – future I	**-22.8**	**<0.001**	**-19.0**	**<0.001**	**-13.2**	**<0.001**
	Present – future II	**-59.7**	**<0.001**	**-40.3**	**<0.001**	**-10.8**	**<0.001**
	Future I – future II	**-36.9**	**<0.001**	**-21.3**	**<0.001**	2.5	0.1
**Southern Alps**	Historical – present	-23.9	0.0508	**-27.3**	**0.019**	**-26.1**	**0.01988**
	Historical – future I	**-59.8**	**<0.001**	**-51.0**	**<0.001**	**-29.4**	**0.00943**
	Historical – future II	**-130.3**	**<0.001**	**-112.3**	**<0.001**	**-39.9**	**<0.001**
	Present – future I	**-35.8**	**0.0033**	-23.7	0.158	-3.3	1.0
	Present – future II	**-106.4**	**<0.001**	**-85.0**	**<0.001**	-13.7	0.64773
	Future I – future II	**-70.5**	**<0.001**	**-61.4**	**<0.001**	-10.5	1.0
