# Supplementary material for: Global reduction of snow cover in ski areas under climate change
Source: PLoS One. 2024 Mar 13;19(3):e0299735. doi: 10.1371/journal.pone.0299735 (PMC10936838; doi:10.1371/journal.pone.0299735)
Supplement: S5 Table — Confidence intervals are given (CI 5;95). Data for very high, high and low emissions scenarios is given. (PDF) [file pone.0299735.s005.pdf]

Region	Timely comparison	SSP5 -8.5<br>% mean change;<br>CI (5;95)	SSP3-7.0<br>% mean change;<br>CI (5;95)	SSP1-2.6<br>% mean change;<br>CI (5;95)
**All regions**	Historical – present	-13.3 (-13.1; -13,4)	-15.2 (-14.9; -15.5)	-15.5 (-15.3; -15.7)
	Historical –future I	-30.2 (-29.8; -30.5)	-28.7 (-28.3; -29.1)	-18.8 (-18.6; -19.1)
	Historical – future II	-45.0 (-44.6; -45.4)	-39.4 (-39.1; -39.8)	-17.3 (-17.1; -17.5)
	Present – future I	-21.1 (-20.6; -21.5)	-18.0 (-17.7; -18.4)	-4.8 (-4.6; -5.0)
	Present – future II	-38.2 (-37.8; -38.6)	-30.4 (-30.1; -30.8)	-1.8 (-1.7; -2.0)
	Future I – future II	-24.6 (-24.2; -25.0)	-16.5 (-16.2; -16.7)	2.3 (2.5; 2.2)
**Andes**	Historical – present	-13.9 (-10.8; -16.9)	-12.4 (-10.0; -14.9)	-12.1 (-9.5; -14.7)
	Historical – future I	-27.8 (-24.3; -31.3)	-32.4 (-28.3; -36.6)	-12.8 (-10.2; -15.7)
	Historical – future II	-49.5 (-45.1; -53.4)	-43.3 (-39.2; -47.7)	-16.9 (-13.6; -20.2)
	Present – future I	-17.8 (-15.2; -20.5)	-25.9 (-21.8; -29.9)	-1.4 (-0.1; -3.0)
	Present – future II	-44.1 (-39.6; -48.5)	-38.5 (-34.2; -42.8)	-6.8 (-4.3; -9.2)
	Future I – future II	-34.7 (-30.0; 39.4)	-19.4 (-16.2; -22.7)	-5.7 (-3.5; -8.0)
**Appalachian**	Historical – present	-13.9 (-13.1; -14.8)	-11.2 (-10.5; -11.8)	-14.7 (-13.9; -15.5)
	Historical – future I	-29.9 (-28.5; -31.3)	-22.9 (21.7; -24.1)	-19.0 (-18.0; -19.9)
	Historical – future II	-50.1 (-48.5; -51.6)	-37.0 (35.6; -38.5)	-21.3 (-20.2; -22.3)
	Present – future I	-19.6 (-18.3; -21.0)	-13.9 (-12.7; -15.0)	-5.6 (-4.9; -6.3)
	Present – future II	-43.0 (-41.3; -44.6)	-29.8 (-28.3; -31.4)	-8.5 (-7.6; -9.4)
	Future I – future II	-31.2 (-29.4; -32.8)	-19.3 (-17.9; -20.6)	-3.4 (-2.9; -4.0)
**Australian Alps**	Historical – present	-16.3 (-13.9; -18.6)	-21.3 (-18.7; -23.9)	-19.3 (-15.4; -23.2)
	Historical – future I	-54.4 (-48.7; -60.2)	-51.3 (-45.6; -57.0)	-30.6 (-25.3; -36.0)
	Historical – future II	-89.1 (-85.4; -92.8)	-78.2 (-73.6; -82.9)	-32.6 (-27.3; 37.9)
	Present – future I	-47.6 (-41.1; -54.0)	-41.6 (-35.0; -48.1)	-16.5 (-11.3; -21.7)
	Present – future II	-87.7 (-83.6; -91.9)	-74.6 (-69.2; -80.0)	-18.9 (-13.8; -24.0)
	Future I – future II	-79.4 (-72.8; -85.9)	-59.7 (-52.4; -66.9)	-2.9 (-2.3; -3.5)
**European Alps**	Historical – present	-14.6 (-14.4; -14.8)	-17.4 (-17.1; -17.8)	-17.2 (-17.0; -17.5)
	Historical – future I	-32.4 (-32.0; -32.8)	-31.6 (-31.1; -32.0)	-19.7 (-19.4; -20.0)
	Historical – future II	-46.4 (-45.9; -46.9)	-41.8 (-41.4; -42.2)	-17.4 (-17.2; -17.6)
	Present – future I	-22.6 (-22.2; -23.0)	-19.6 (-19.1; -20.0)	-4.0 (-3.8; -4.3)
	Present – future II	-39.1 (-38.6; -39.6)	-31.7 (-31.3; -32.0)	0.3 (0.5; 0.2)
	Future I – future II	-24.5 (-24.1; -25.0)	-16.3 (-16.1; -16.5)	3.7 (4.0; 3.5)
**Japanese Alps**	Historical – present	-12.8 (-11.9; -13.8)	-15.3 (-14.1; -16.5)	-17.8 (-16.5; -19.1)
	Historical – future I	-37.4 (-35.8; -39.1)	-35.1 (-33.3; -36.9)	-24.5 (-23.0; 26.1)
	Historical – future II	-51.7 (-50.0; 53.5)	-49.7 (-47.9; -51.4)	-24.2 (-22.9; -25.6)
	Present – future I	-30.1 (-28.4; -31.8)	-25.5 (-23.7; -27.2)	-9.8 (-8.7; -11.0)
	Present – future II	-46.3 (-44.5; -48.2)	-42.6 (-40.7; -44.4)	-8.9 (-8.1; -9.6)
	Future I – future II	-26.9 (-25.1; -28.8)	-25.6 (-24.0; -27.2)	-1.3 (-1.0; -1.6)
**Rocky Mountains**	Historical – present	-6.8 (-6.5; -7.2)	--6.7 (-6.4; -7.0)	-7.1 (-6.7; -7.4)
	Historical – future I	-15.9 (-15.3; -16.4)	-14.3 (-13.9; -14.8)	-12.2 (-11.7; 12.6)
	Historical – future II	-30.5 (-30.0; -31.4)	-22.7 (-22.1; -23.4)	-11.2 (-10.7; -11.6)
	Present – future I	-10.1 (-9.6; -10.6)	-8.5 (-8.0; -8.9)	-5.8 (-5.4; -6.1)
	Present – future II	-25.9 (-24.9; -26.8)	-17.5 (-16.9; -18.2)	-4.7 (-4.3; -5.0)
	Future I – future II	-18.6 (-17.7; -19.5)	-10.7 (-10.1; -11.2)	1.1 (1.2; 0.9)
**Southern Alps**	Historical – present	-10.4 (-8.2; -12.5)	-11.9 (-9.7; -14.0)	-10.8 (-10.0; -11.6)
	Historical – future I	-27.4 (-22.9; -31.8)	-23.1 (-19.5; -26.7)	-12.9 (10.9; 15.0)
	Historical – future II	-59.2 (-53.0; -65.5)	-50.9 (-45.4; -56.3)	-17.5 (-15.3; -19.6)
	Present – future I	-19.1 (-14.5; -23.7)	-13.0 (-9.6; 16.5)	-2.6 (-0.3; -5.0)
	Present – future II	-54.5 (-47.6; 61.4)	-44.5 (-38.4; -50.5)	-7.8 (-5.4; -10.1)
	Future I – future II	-45.8 (-38.3; -53.4)	-38.4 (-32.1; -44.8)	-5.4 (-4.8; -5.9)
