# Supplementary figures and images for: Global reduction of snow cover in ski areas under climate change
Source: PLoS One. 2024 Mar 13;19(3):e0299735. doi: 10.1371/journal.pone.0299735 (PMC10936838; doi:10.1371/journal.pone.0299735)

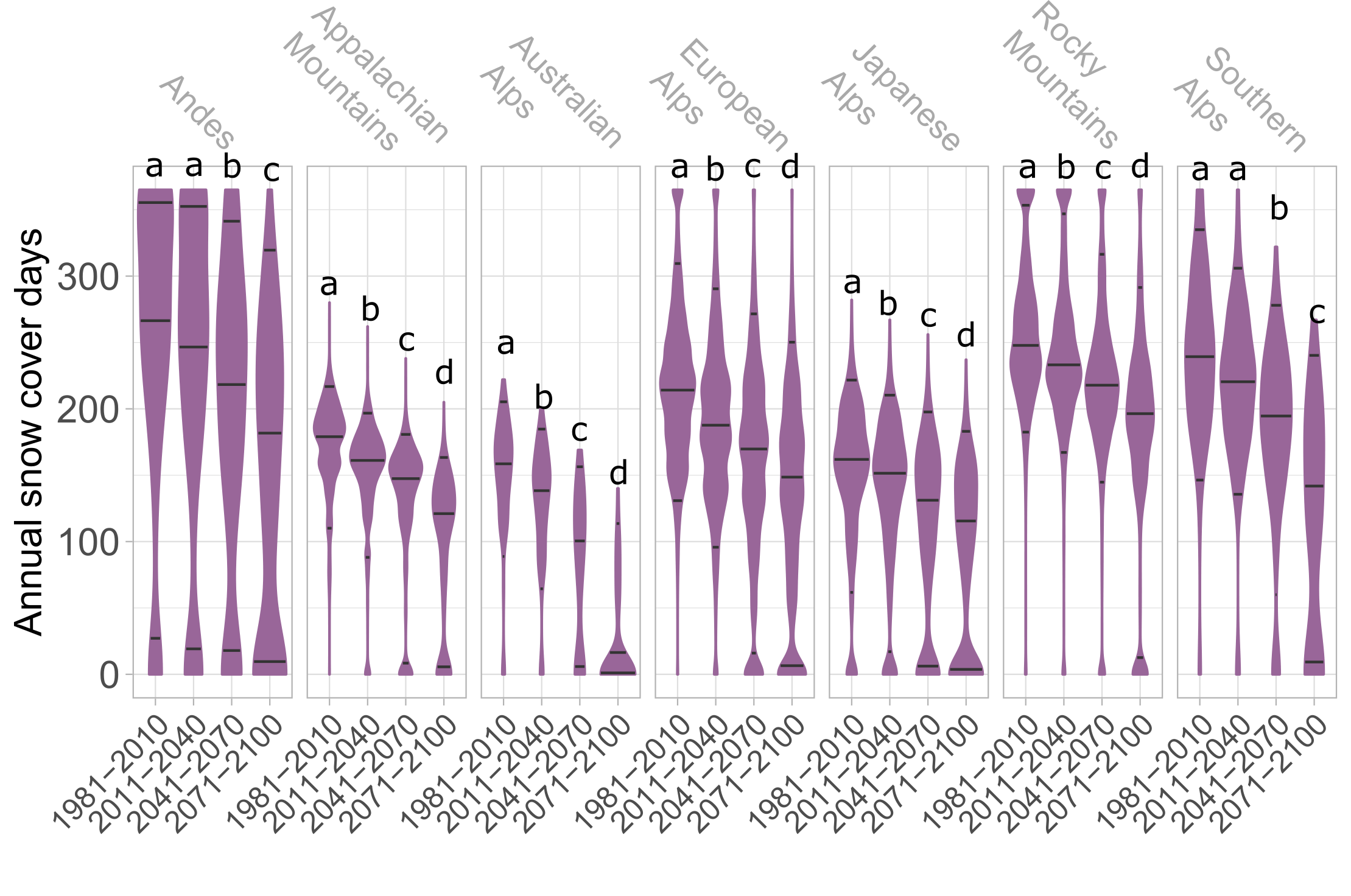

Supplement: S1 Fig — The median and the 5%;95% confidence intervals are indicated by black lines. Letters above the violin plots indicate significant differences between groups (Kruskal-Wallis test, Pairwise Wilcoxon test with Bonferroni correction, e.g. a differs significantly from b, c, and d). (TIF) [file pone.0299735.s006.tif]

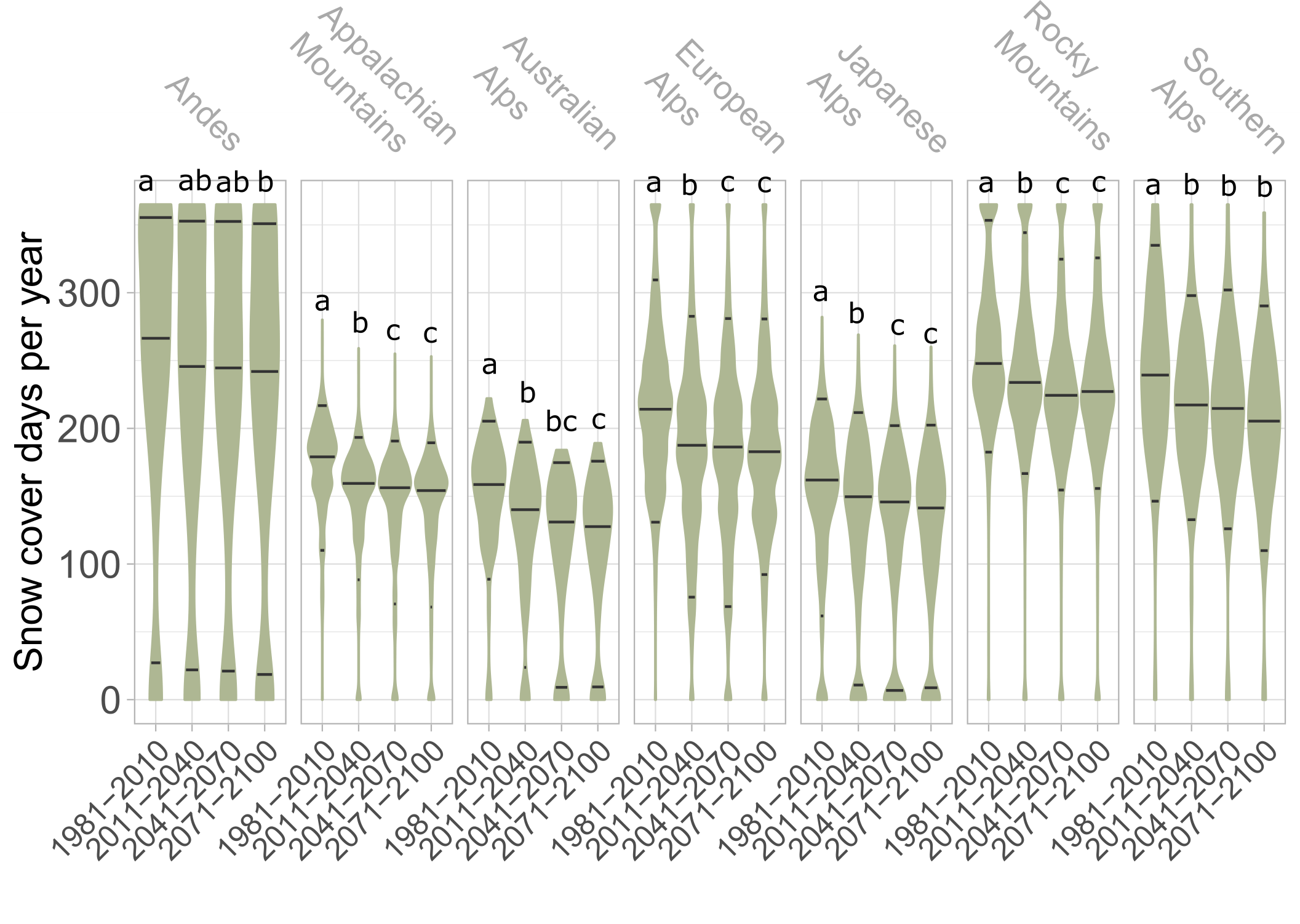

Supplement: S2 Fig — The median and the 5%;95% confidence intervals are indicated by black lines. Letters above the violin plots indicate significant differences between groups (Kruskal-Wallis test, Pairwise Wilcoxon test with Bonferroni correction, e.g. a differs significantly from b, c, and d). (TIF) [file pone.0299735.s007.tif]

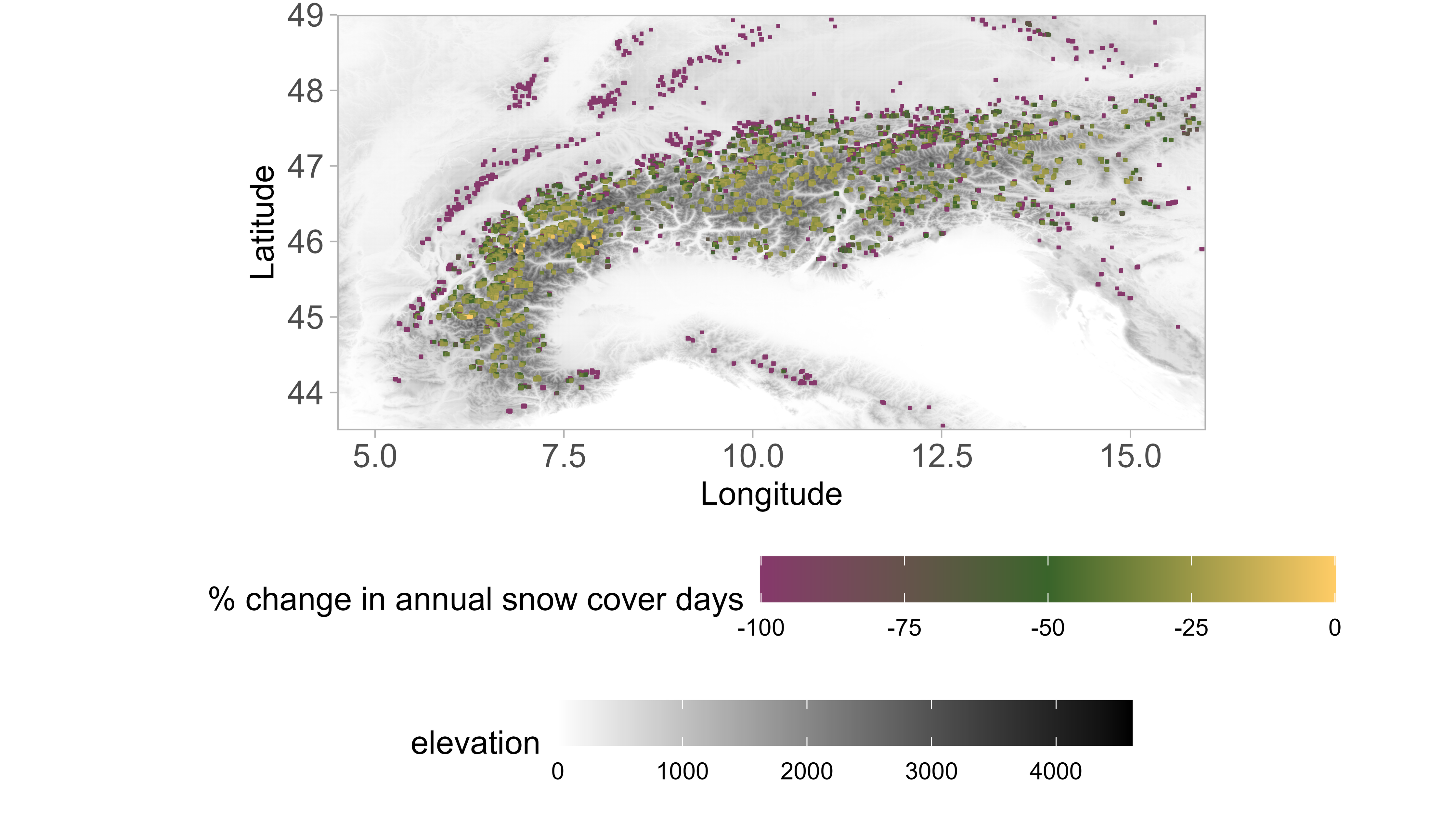

Supplement: S3 Fig — Colouring indicates the percent change in annual snow cover days in each ski area in the future 2071–2100 compared to historical (1981–2010) snow cover days under very high emissions (SSP5-8.5) scenario. (TIF) [file pone.0299735.s008.tif]

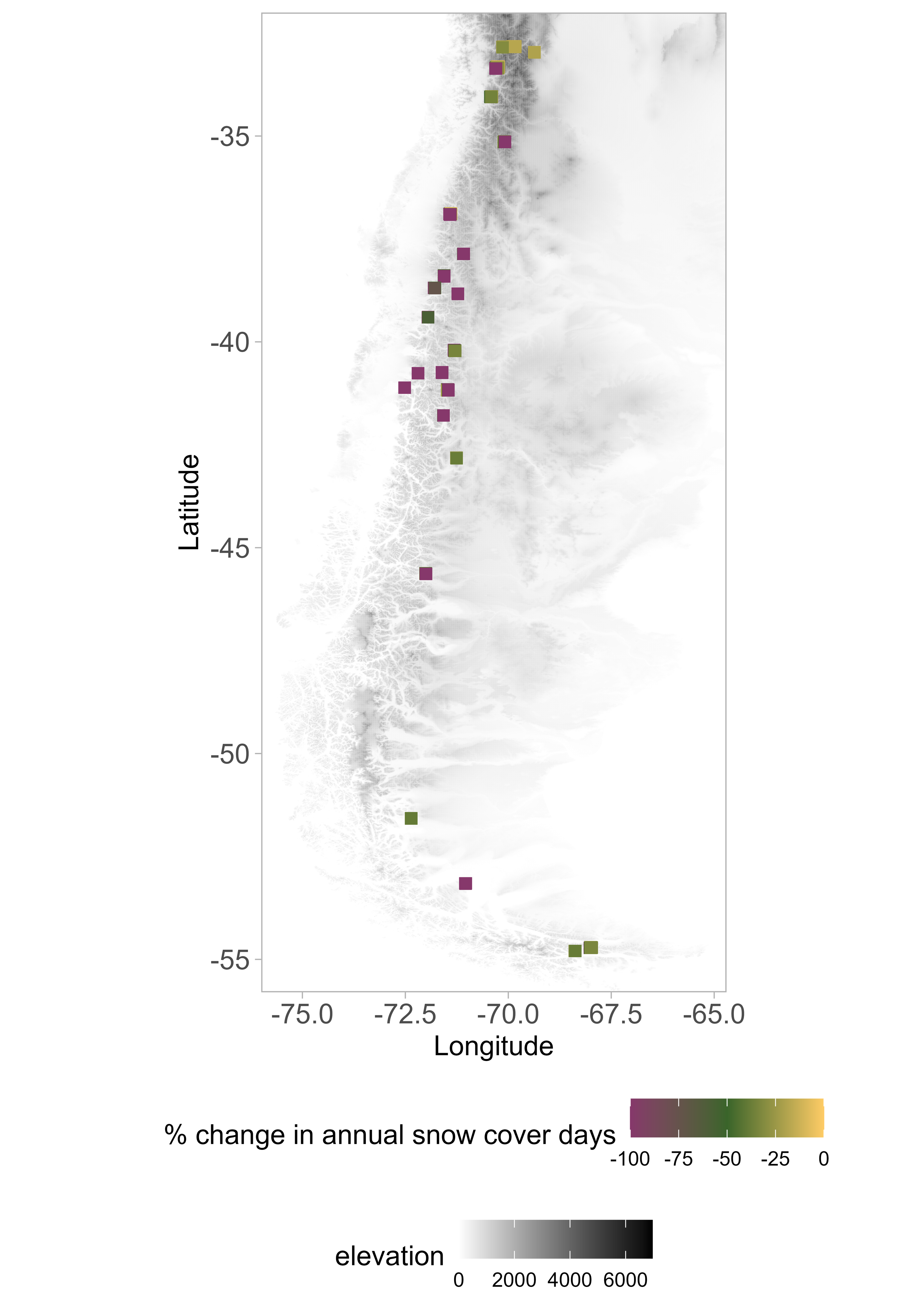

Supplement: S4 Fig — Colouring indicates the percent change in annual snow cover days in each ski area in the future 2071–2100 compared to historical (1981–2010) snow cover days under very high emissions (SSP5-8.5) scenario. (TIF) [file pone.0299735.s009.tif]

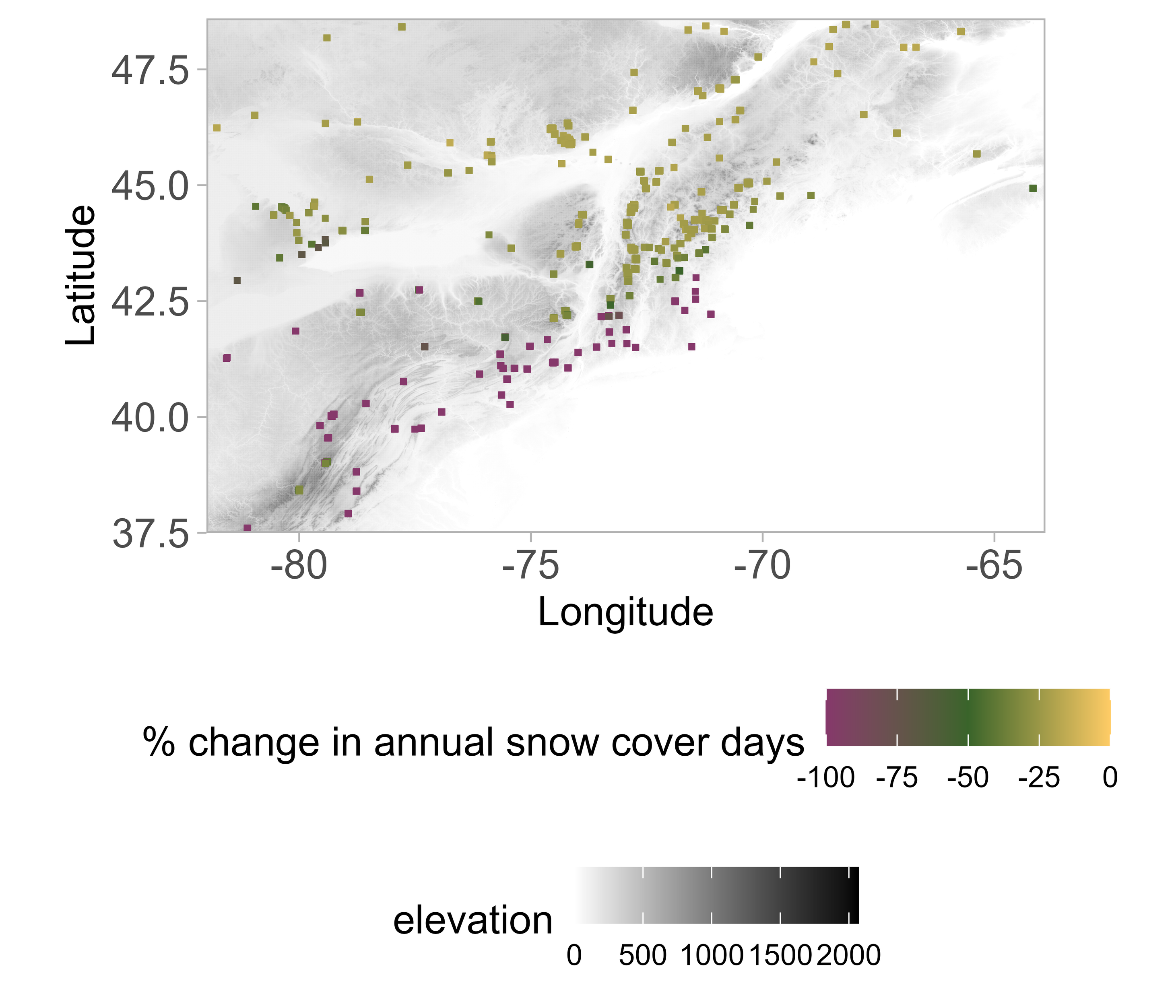

Supplement: S5 Fig — Colouring indicates the percent change in annual snow cover days in each ski area in the future 2071–2100 compared to historical (1981–2010) snow cover days under very high emissions (SSP5-8.5) scenario. (TIF) [file pone.0299735.s010.tif]

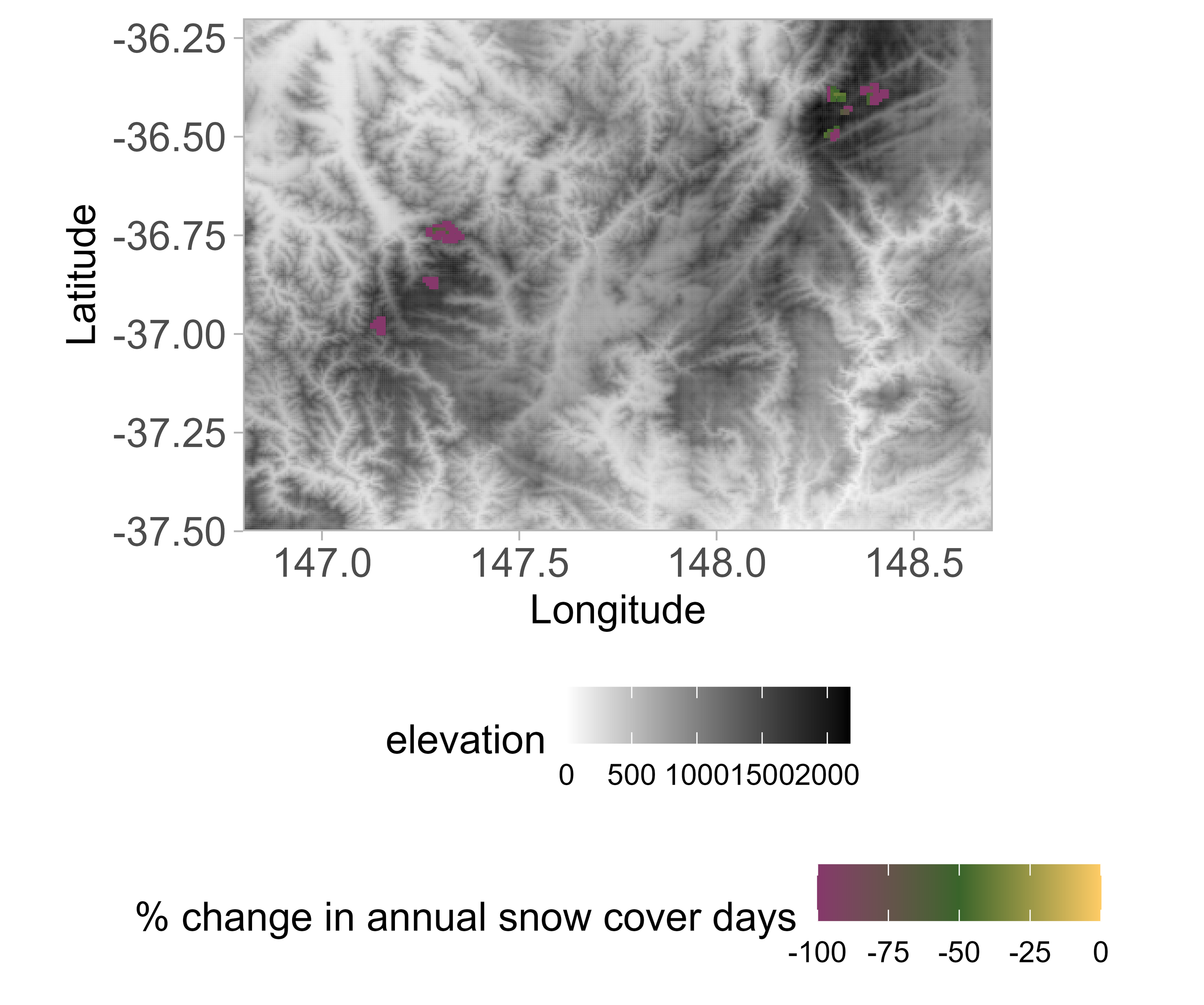

Supplement: S6 Fig — Colouring indicates the percent change in annual snow cover days in each ski area in the future 2071–2100 compared to historical (1981–2010) snow cover days under very high emissions (SSP5-8.5) scenario. (TIF) [file pone.0299735.s011.tif]

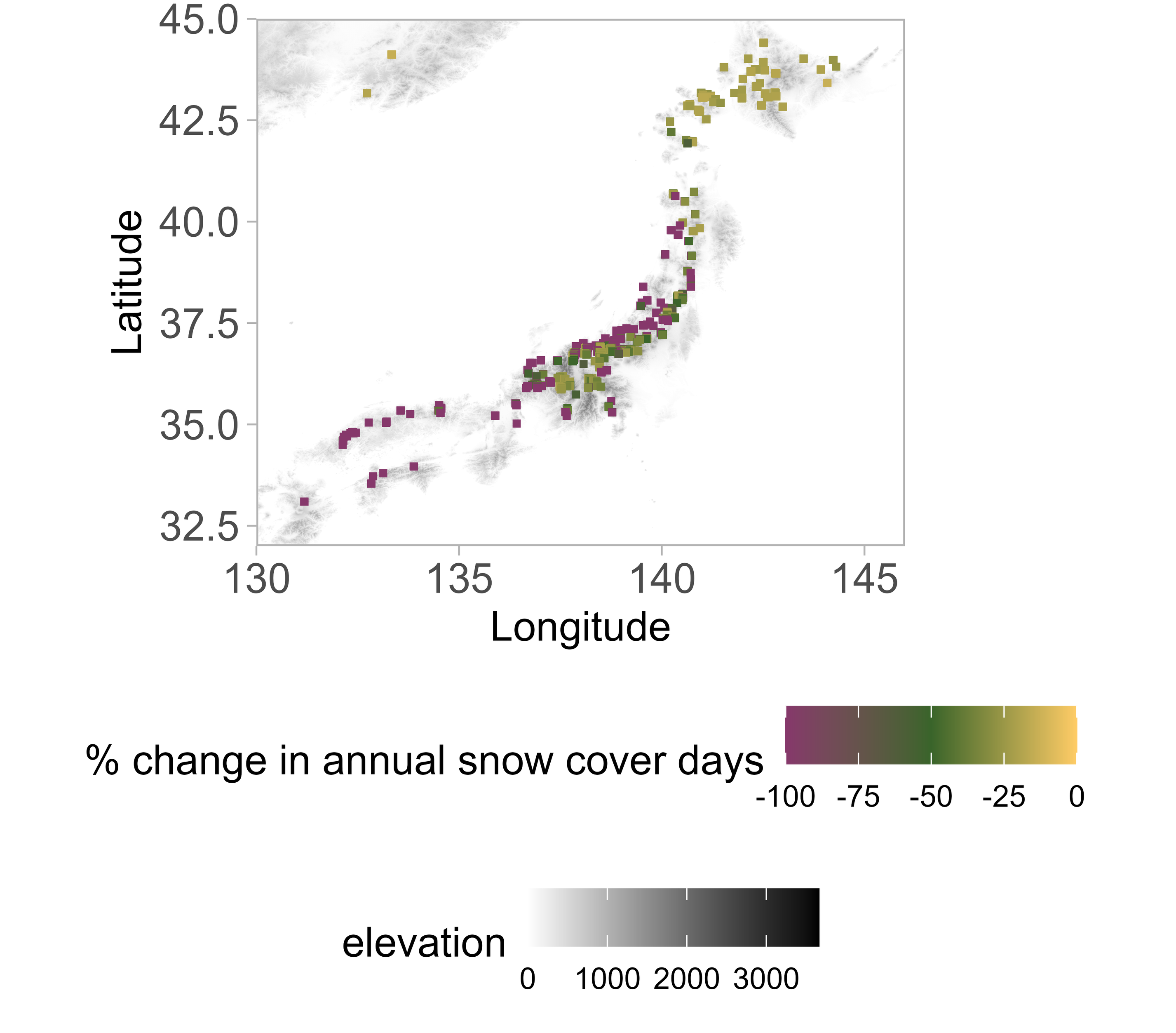

Supplement: S7 Fig — Colouring indicates the percent change in annual snow cover days in each ski area in the future 2071–2100 compared to historical (1981–2010) snow cover days under very high emissions (SSP5-8.5) scenario. (TIF) [file pone.0299735.s012.tif]

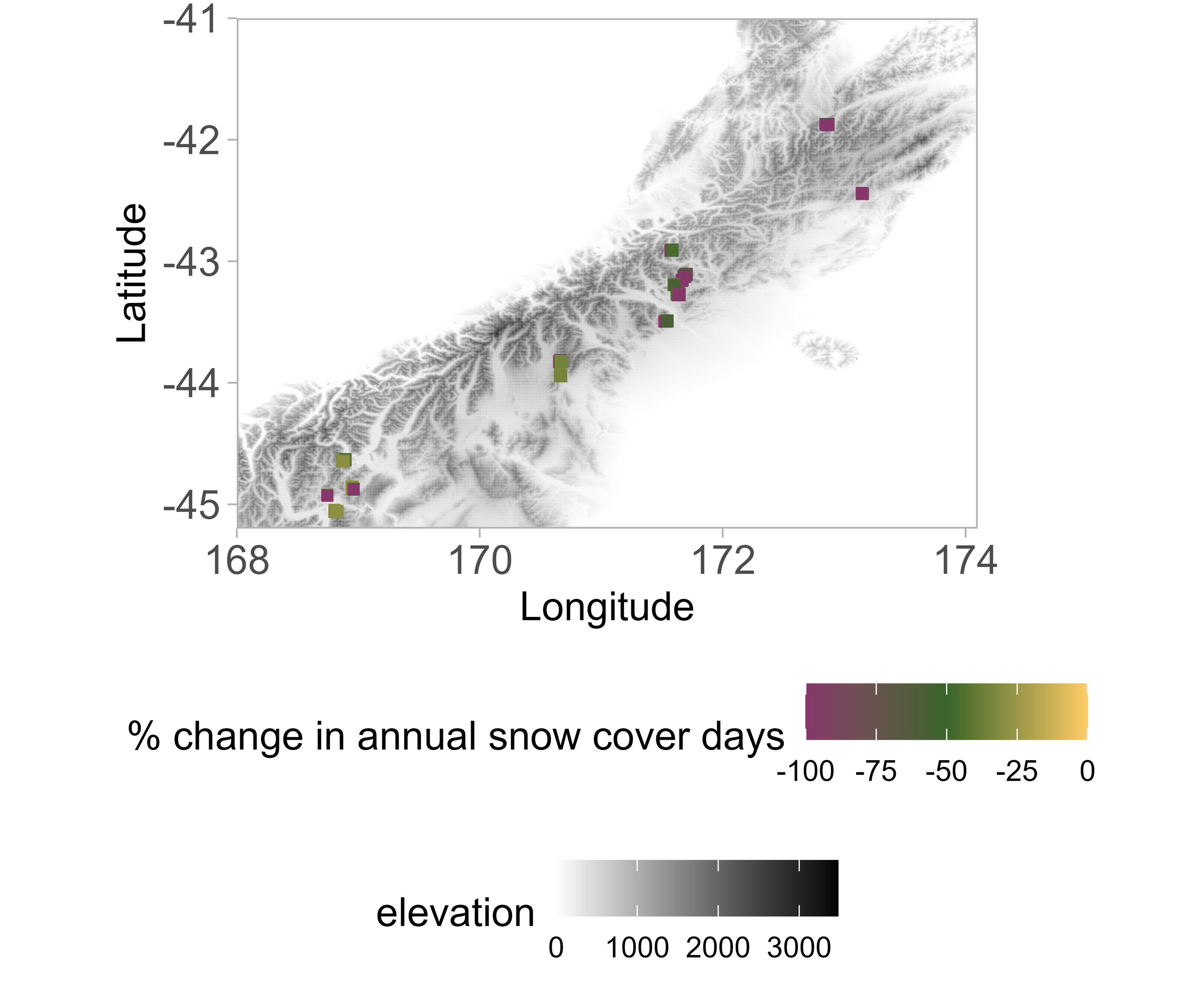

Supplement: S8 Fig — Colouring indicates the percent change in annual snow cover days in each ski area in the future 2071–2100 compared to historical (1981–2010) snow cover days under very high emissions (SSP5-8.5) scenario. (TIF) [file pone.0299735.s013.tif]

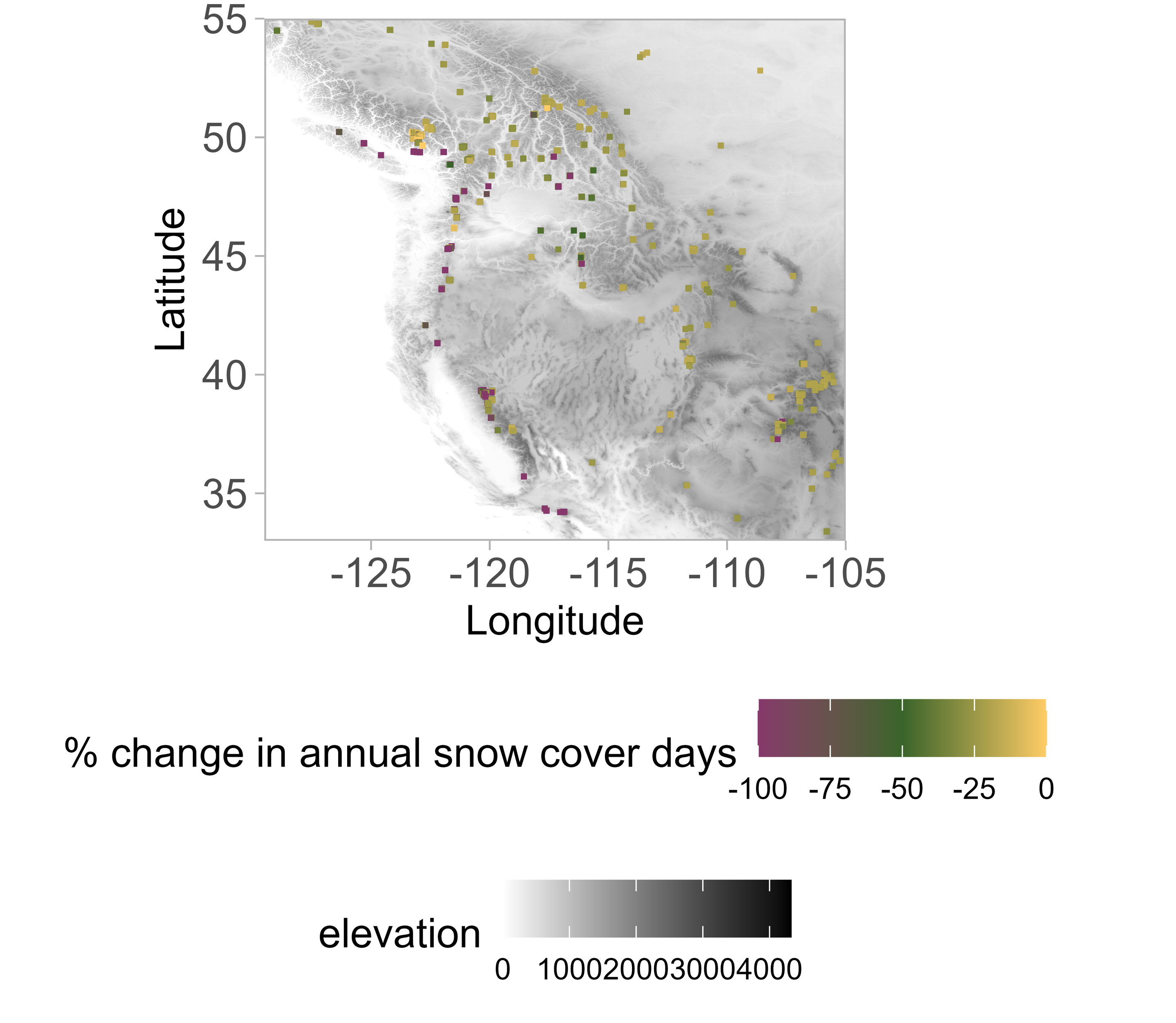

Supplement: S9 Fig — Colouring indicates the percent change in annual snow cover days in each ski area in the future 2071–2100 compared to historical (1981–2010) snow cover days under very high emissions (SSP5-8.5) scenario. (TIF) [file pone.0299735.s014.tif]

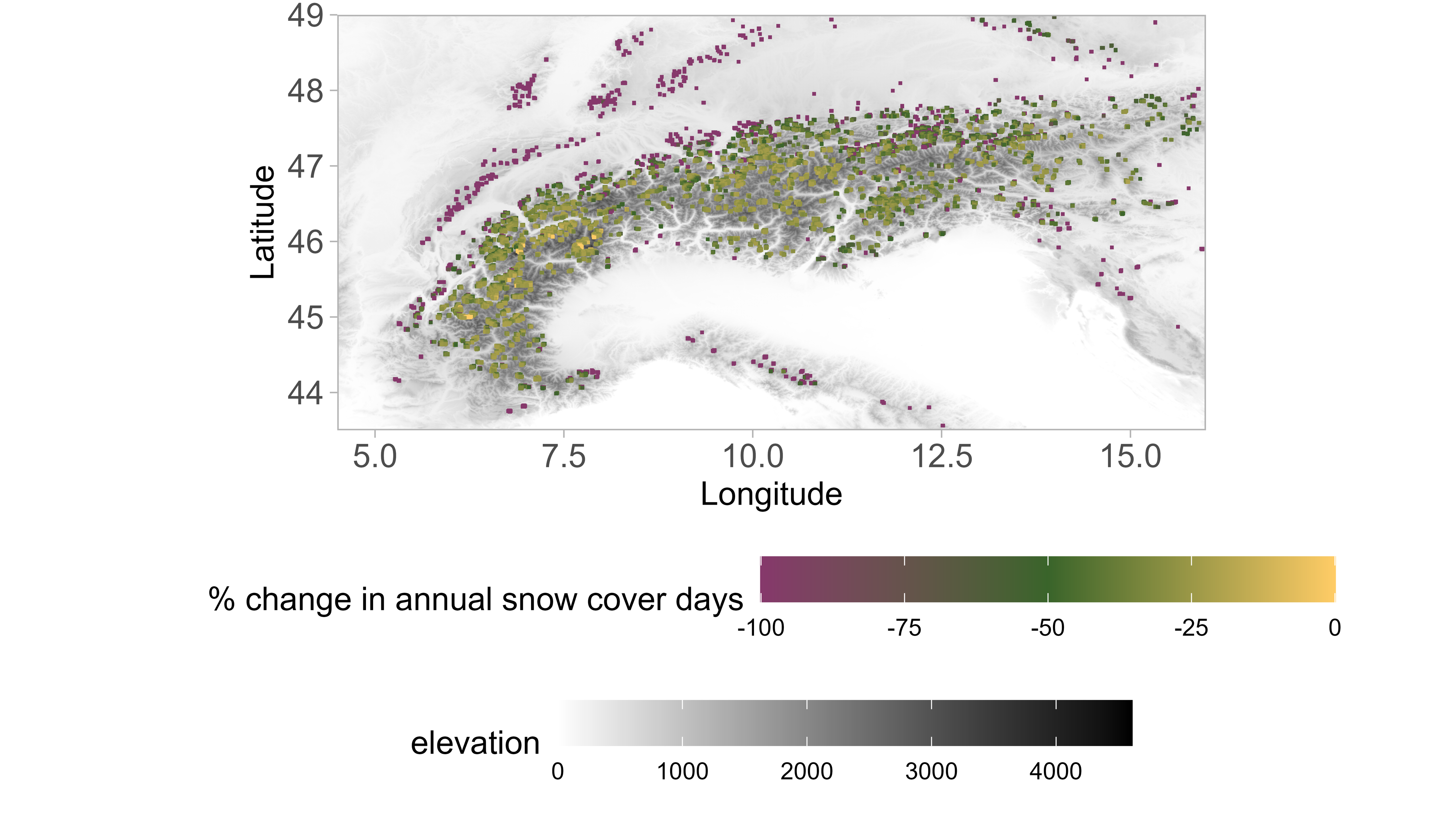

Supplement: S10 Fig — Colouring indicates the percent change in annual snow cover days in each ski area in the future 2071–2100 compared to historical (1981–2010) snow cover days under high emissions (SSP3-7.0) scenario. (TIF) [file pone.0299735.s015.tif]

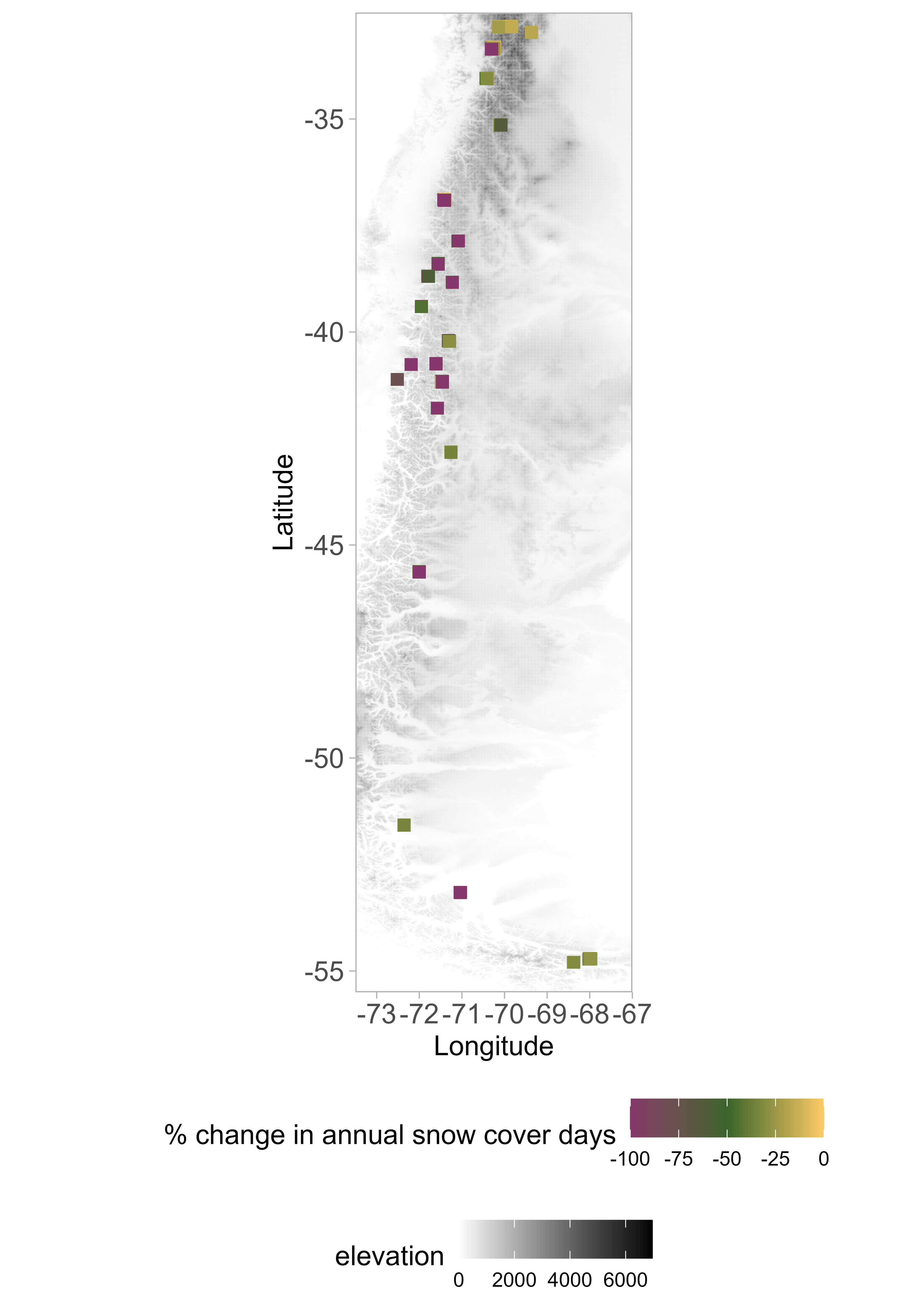

Supplement: S11 Fig — Colouring indicates the percent change in annual snow cover days in each ski area in the future 2071–2100 compared to historical (1981–2010) snow cover days under high emissions (SSP3-7.0) scenario. (TIF) [file pone.0299735.s016.tif]

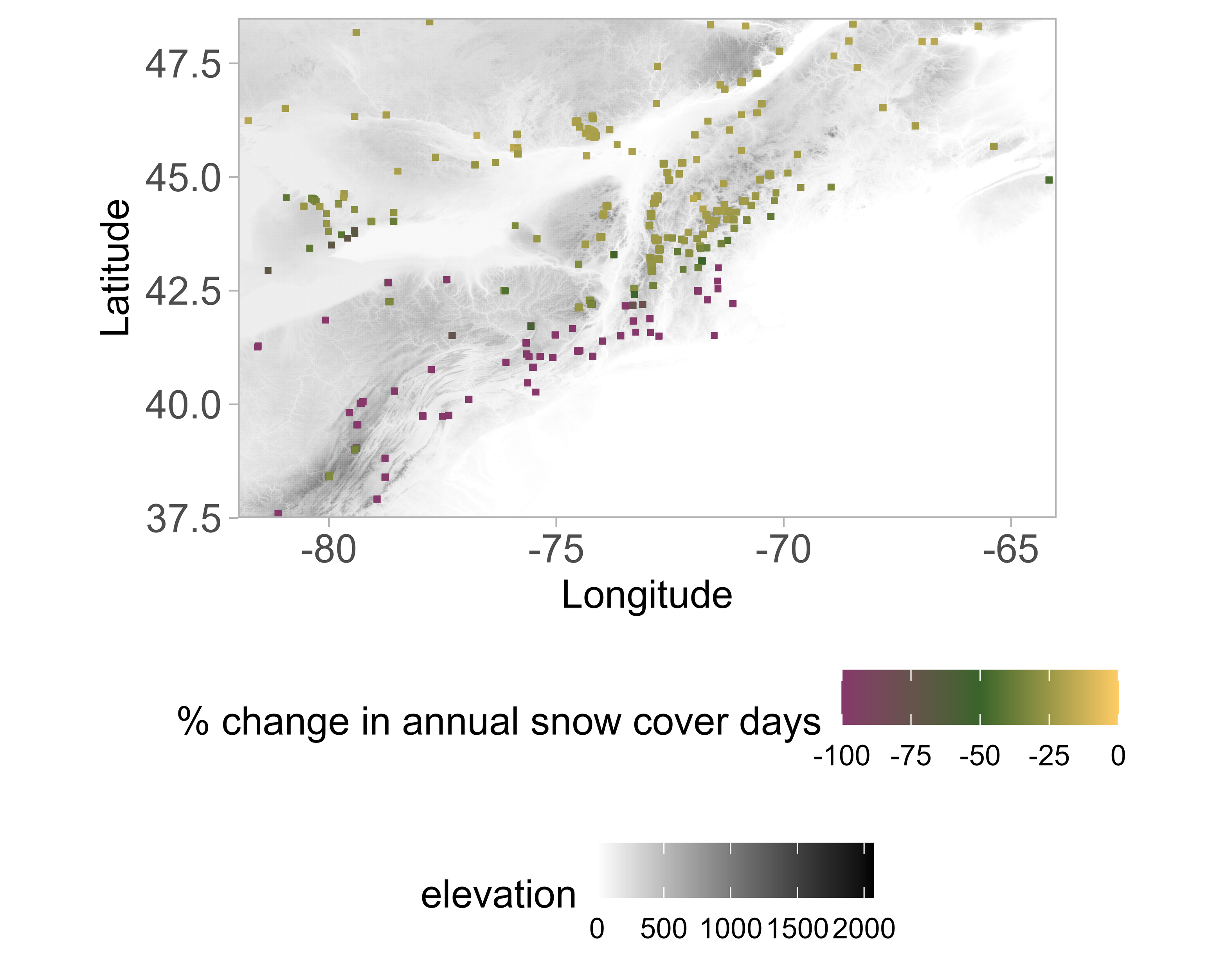

Supplement: S12 Fig — Colouring indicates the percent change in annual snow cover days in each ski area in the future 2071–2100 compared to historical (1981–2010) snow cover days under high emissions (SSP3-7.0) scenario. (TIF) [file pone.0299735.s017.tif]

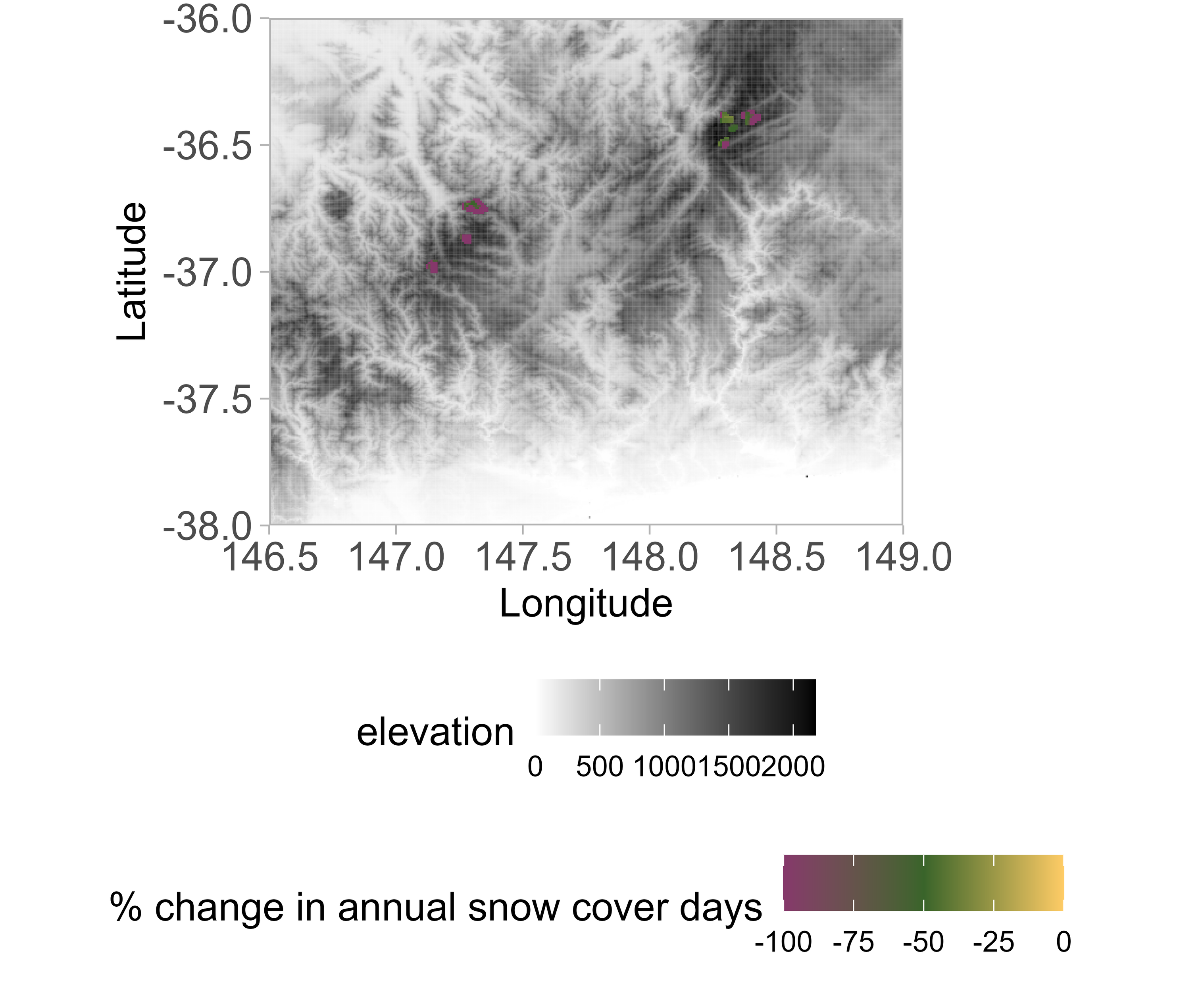

Supplement: S13 Fig — Colouring indicates the percent change in annual snow cover days in each ski area in the future 2071–2100 compared to historical (1981–2010) snow cover days under high emissions (SSP3-7.0) scenario. (TIF) [file pone.0299735.s018.tif]

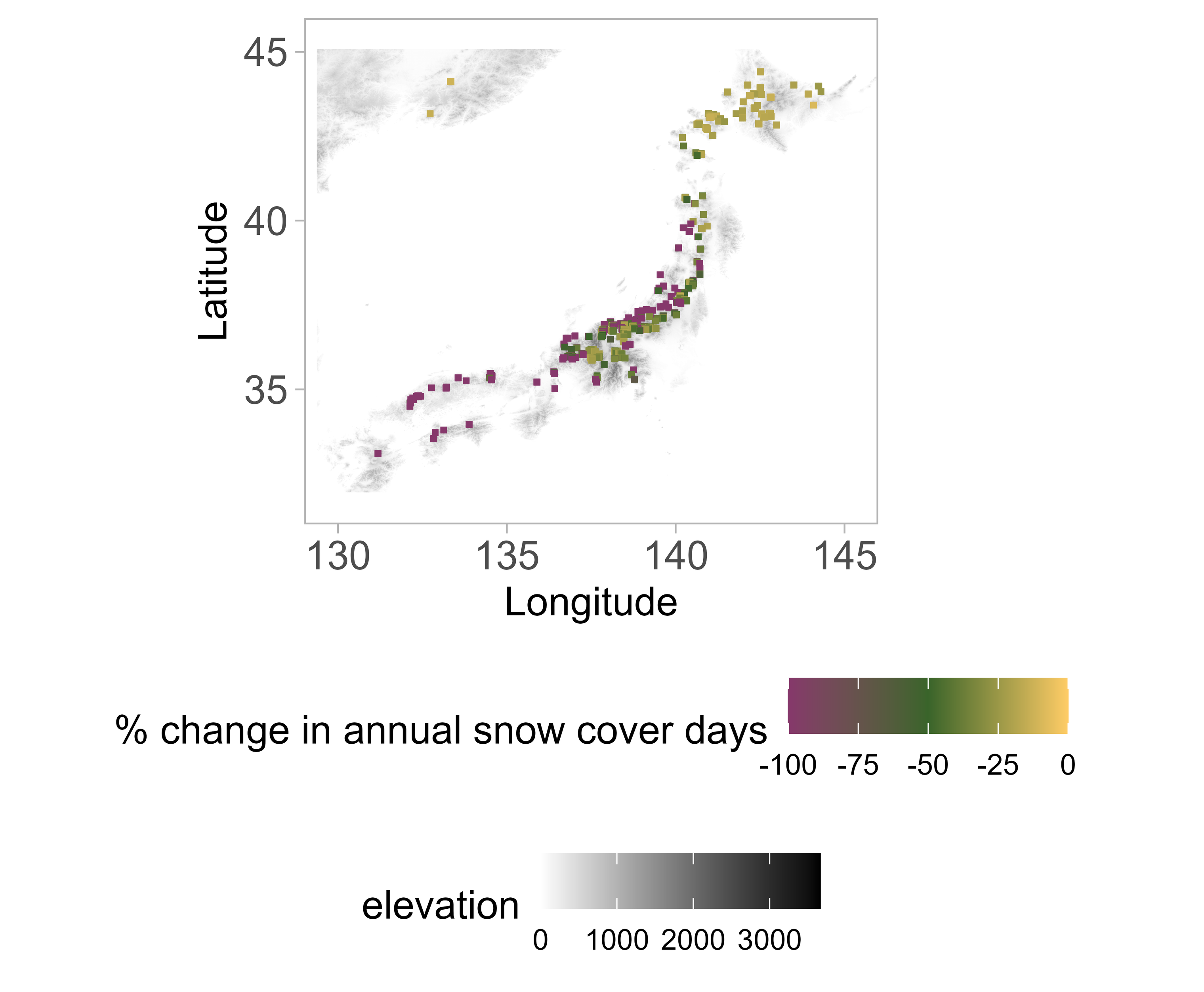

Supplement: S14 Fig — Colouring indicates the percent change in annual snow cover days in each ski area in the future 2071–2100 compared to historical (1981–2010) snow cover days under high emissions (SSP3-7.0) scenario. (TIF) [file pone.0299735.s019.tif]

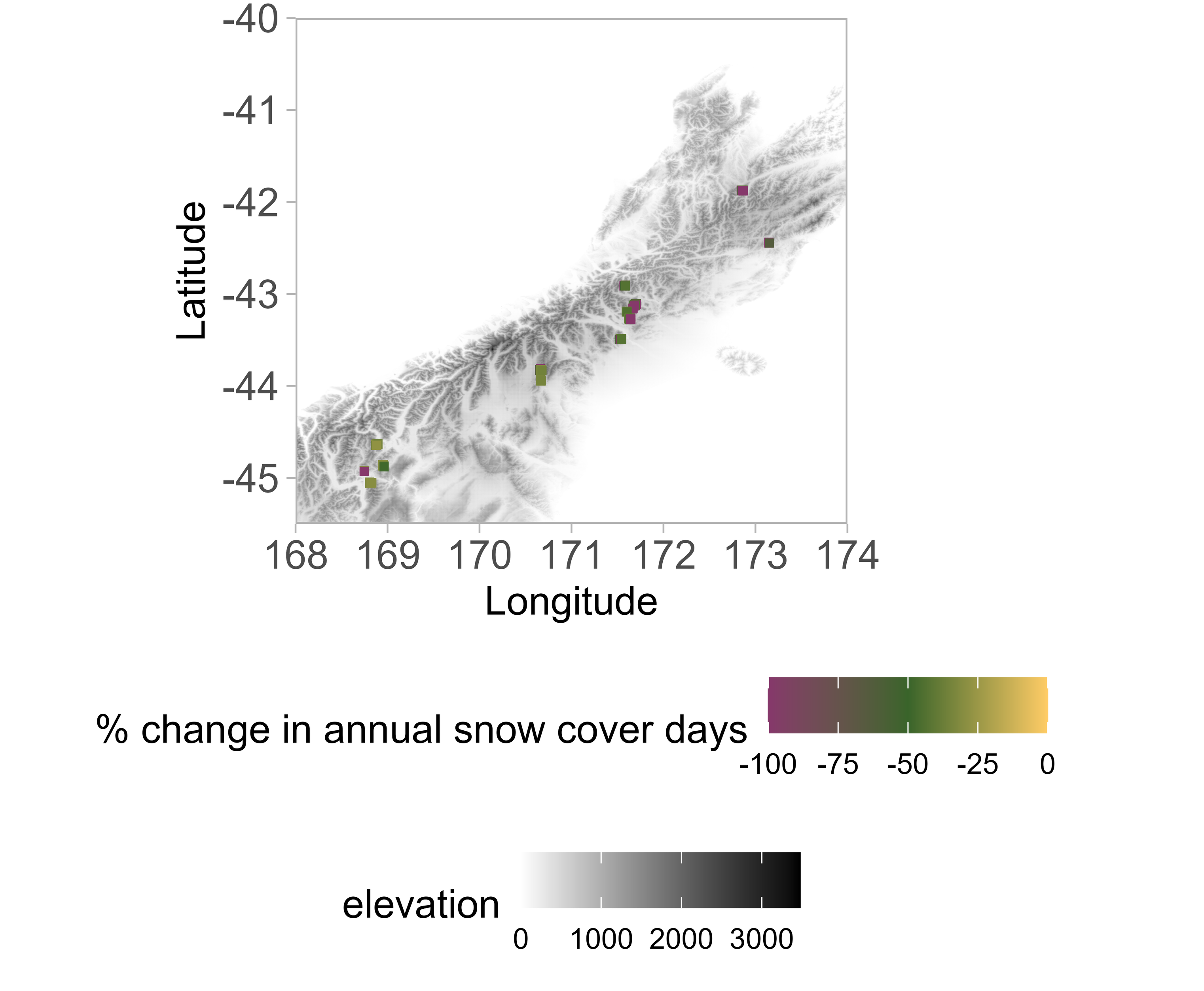

Supplement: S15 Fig — Colouring indicates the percent change in annual snow cover days in each ski area in the future 2071–2100 compared to historical (1981–2010) snow cover days under high emissions (SSP3-7.0) scenario. (TIF) [file pone.0299735.s020.tif]

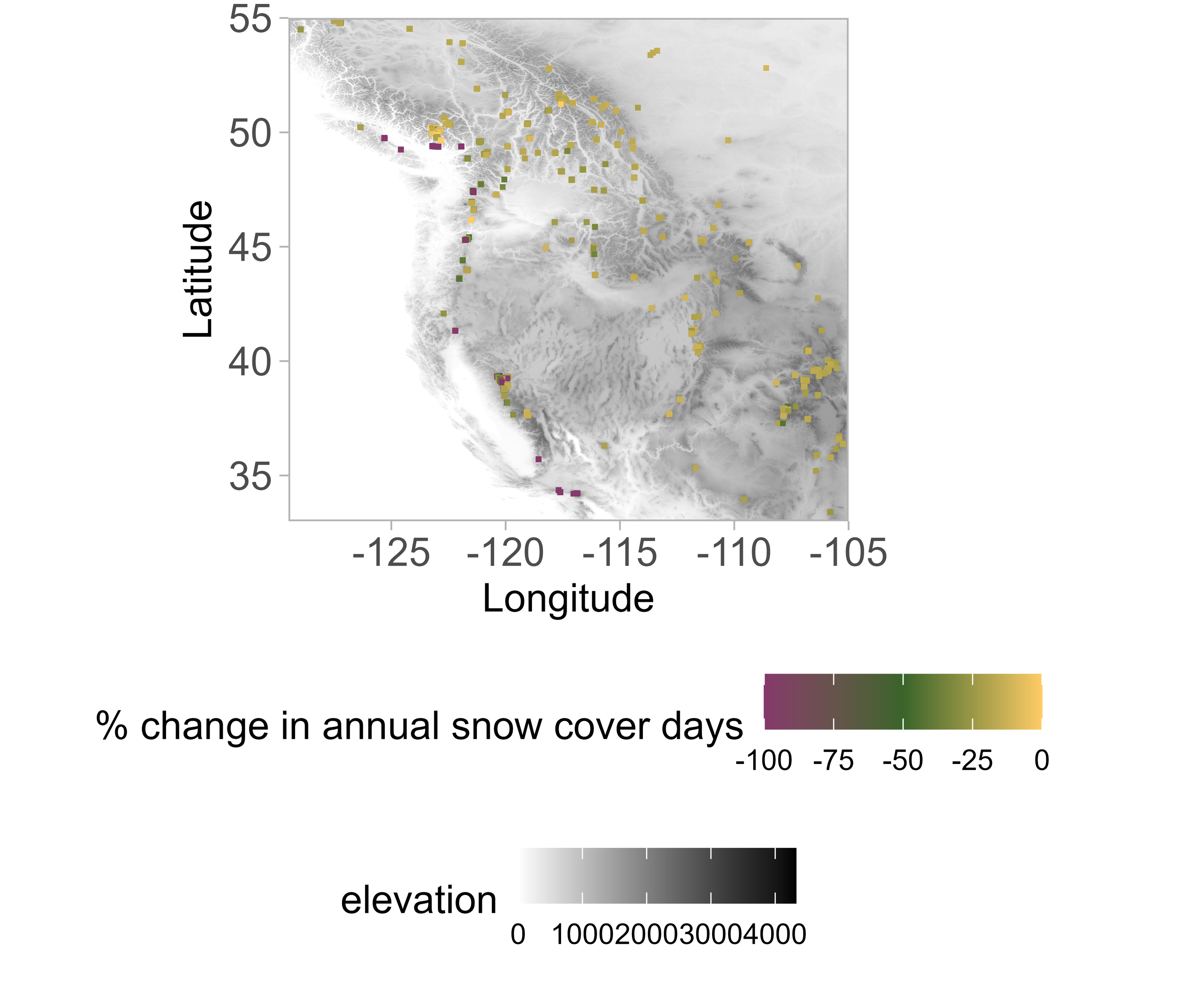

Supplement: S16 Fig — Colouring indicates the percent change in annual snow cover days in each ski area in the future 2071–2100 compared to historical (1981–2010) snow cover days under high emissions (SSP3-7.0) scenario. (TIF) [file pone.0299735.s021.tif]

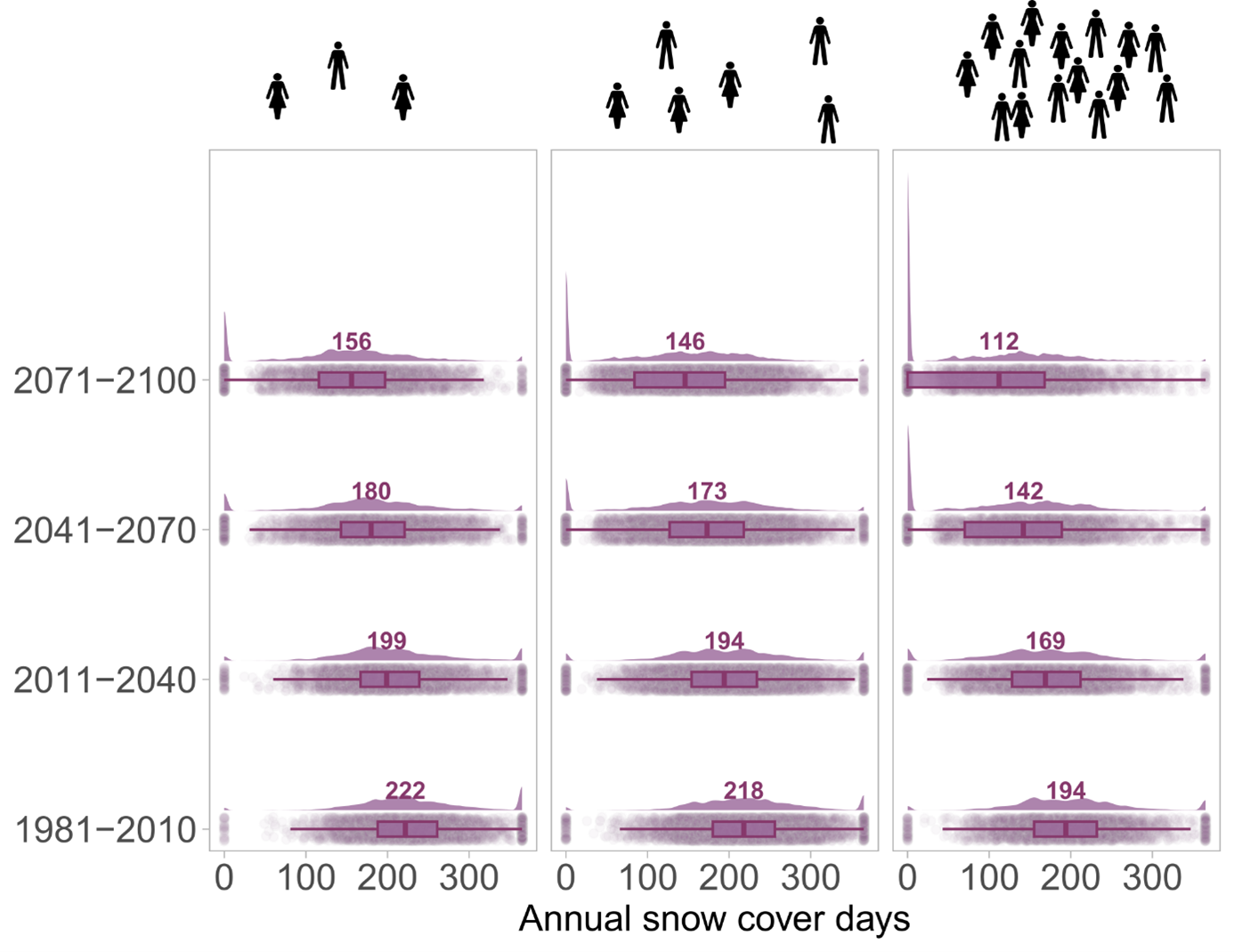

Supplement: S17 Fig — The figure is based on a very high (SSP5-8.5) emissions scenario. (TIF) [file pone.0299735.s022.tif]

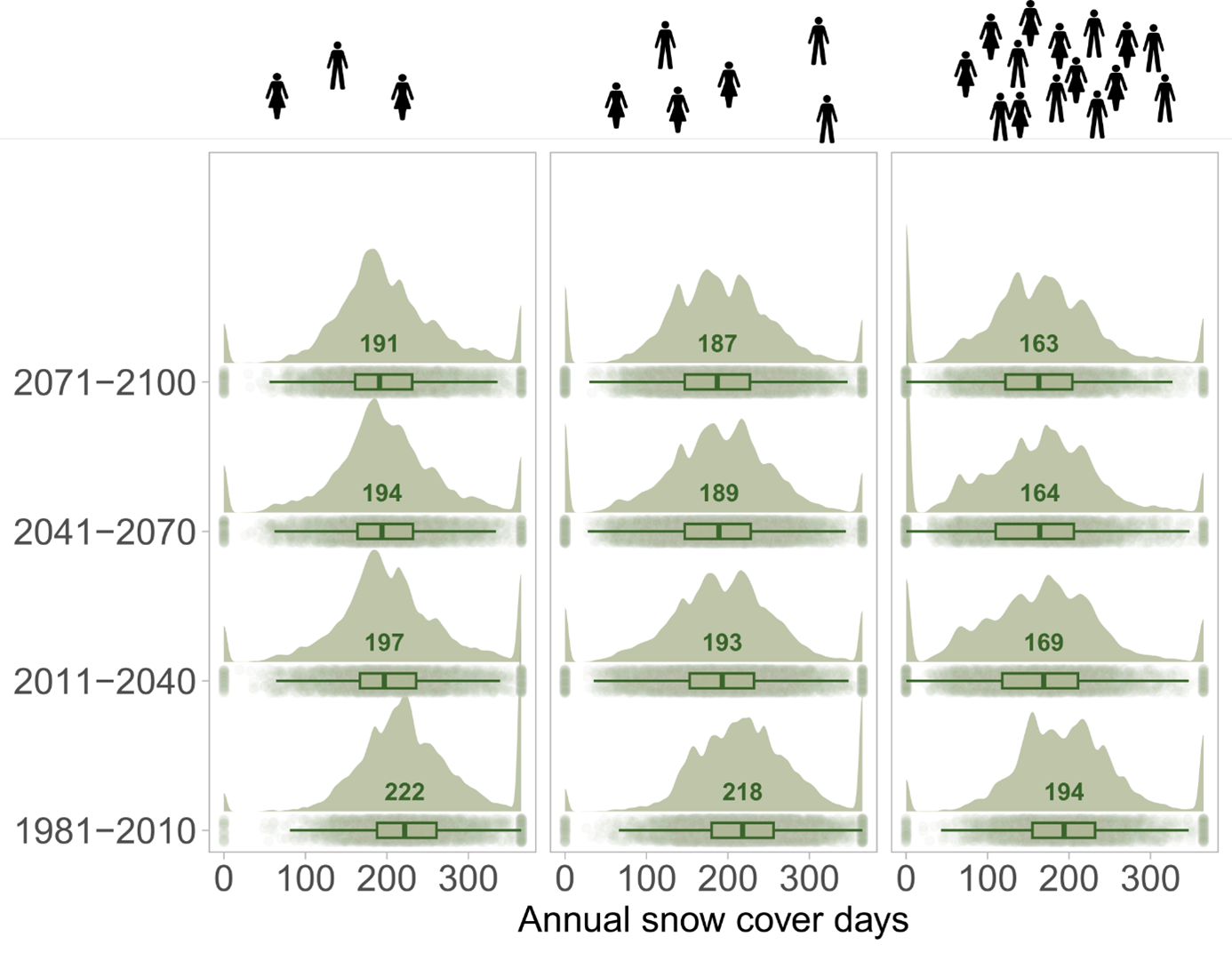

Supplement: S18 Fig — The figure is based on a low (SSP1-2.6) emissions scenario. (TIF) [file pone.0299735.s023.tif]
